# Deposition of MoSe_2_ flakes using cyclic selenides†

**DOI:** 10.1039/d0ra10239c

**Published:** 2021-06-23

**Authors:** Jaroslav Charvot, Raul Zazpe, Richard Krumpolec, Jhonatan Rodriguez-Pereira, David Pavliňák, Daniel Pokorný, Milan Klikar, Veronika Jelínková, Jan M. Macak, Filip Bureš

**Affiliations:** Institute of Organic Chemistry and Technology, Faculty of Chemical Technology, University of Pardubice Studentská 573 Pardubice 53210 Czech Republic filip.bures@upce.cz; Center of Materials and Nanotechnologies, Faculty of Chemical Technology, University of Pardubice Nám. Čs. Legií 565 Pardubice 53002 Czech Republic; Central European Institute of Technology, Brno University of Technology Purkyňova 123 Brno 61200 Czech Republic; Department of Physical Electronics, CEPLANT—R&D Center for Plasma and Nanotechnology Surface Modifications, Faculty of Science, Masaryk University Kotlářská 267/2 61137 Brno Czech Republic; The Institute of Technology and Business in České Budějovice Okružní 517/10 370 01 České Budějovice Czech Republic

## Abstract

The currently limited portfolio of volatile organoselenium compounds used for atomic layer deposition (ALD) has been extended by designing and preparing a series of four-, five- and six-membered cyclic silylselenides. Their fundamental properties were tailored by alternating the ring size, the number of embedded Se atoms and the used peripheral alkyl chains. In contrast to former preparations based on formation of sodium or lithium selenides, the newly developed synthetic method utilizes a direct and easy reaction of elemental selenium with chlorosilanes. Novel 2,2,4,4-tetraisopropyl-1,3,2,4-diselenadisiletane, which features good trade-off between chemical/thermal stability and reactivity, has been successfully used for gas-to-solid phase reaction with MoCl_5_ affording MoSe_2_. A thorough characterization of the as-deposited 2D MoSe_2_ flakes revealed its out-of-plane orientation and high purity. Hence, the developed four-membered cyclic silylselenide turned out to be well-suited Se-precursor for ALD of MoSe_2_.

## Abstract 2

Diselenadisiletanes possess easy preparation, tailored stability, reactivity, volatility and fast exchange ALD reaction to afford MoSe_2_ flakes of high quality.

## Graphical abstract

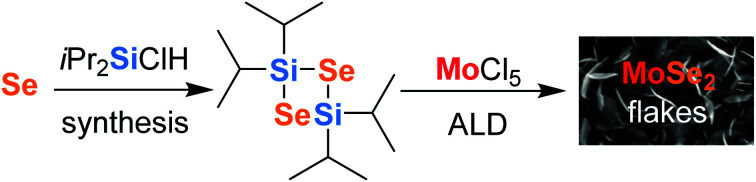

## Introduction

Despite having been developed more than 50 years ago, Atomic Layer Deposition (ALD), a thin-film manufacturing technology,^1^ still attracts growing attention worldwide.^2^ This is due to ALD's unique advantages including high uniformity of prepared nanolayers,^3^ precise thickness control^4^ or possibility to cover non-planar substrates like nanotubes.^5^ A reaction between a gaseous precursor and free functional groups of the selected substrate ensures accurate deposition of atomic monolayer with minimum of defects. On the contrary, exclusive gas-to-solid phase reaction is also one of the biggest ALD limitation. For such reaction, a precursor of sufficient volatility and thermal stability with persisting high reactivity is essential. Finding a compromise between the aforementioned properties is usually not a simple task.

Transition metal dichalcogenides (TMDC) form layered crystal structures featuring chalcogen–metal–chalcogen units bound *via* covalent bonds. Stoichiometric MX_2_ monolayers interacts *via* weak van der Waals forces dependent on the selected metal (M), chalcogen (X) and their supramolecular arrangement.^6^ TMDC are often semiconductors with a narrow band gap – an interesting ability exploitable in electronics, electrocatalysis or photocatalysis, especially in water splitting or hydrogen evolution reactions (HER). In addition to widely explored performance of MoS_2_ in the HER,^7,8^ MoSe_2_,^9^ GaSe (ref. 10) or WSe_2_ (ref. 11) showed also promising results. Bis(trialkylsilyl)selenides^12^ are currently the most favourite ALD selenium precursors used for deposition of the latter selenides. Recently, selenium dimethyldithiocarbamate was successfully used for deposition of Sb_2_Se_3_ as presented by Sarkar.^13^ Our research group introduced bis(trialkylstanyl)selenides^14^ and cyclic silylselenides^15^ as Se-precursors with decreased sensitivity towards air and moisture. Six-membered selenide containing two selenium atoms turned out to be the best precursor so far. This prompted us to explore the family of cyclic silylselenides bearing more selenium atoms further.

## Results and discussion

### Synthesis

**Scheme 1:**
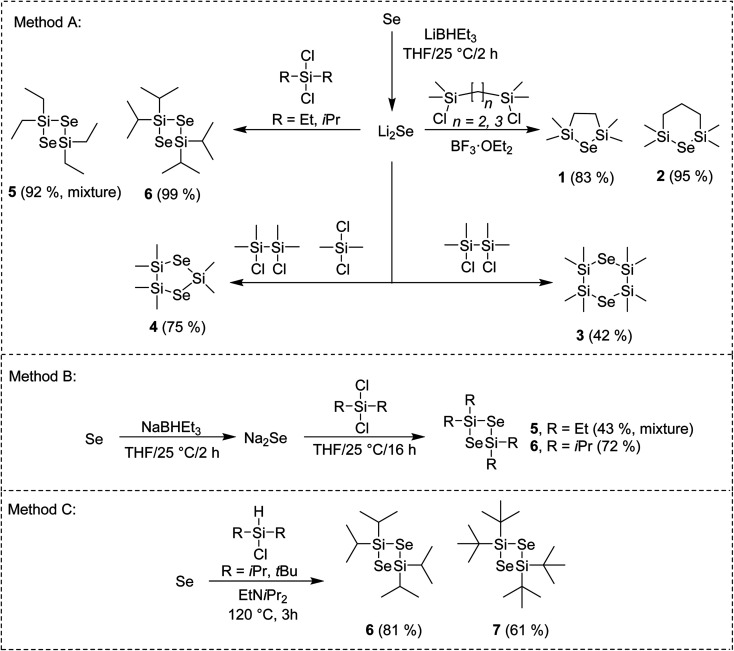
Reaction pathway towards cyclic silylselenides.

The general reaction pathway towards cyclic silylselenides is outlined in Scheme 1. The synthesis and thermal properties of 1 (ref. 16) 2 (ref. 17) and 3 (ref. 17) were reported earlier, see also our recent communication for comprehensive characterization.^15^ The general methodology utilizes Li_2_Se, prepared from elemental Se and its reaction with Li or LiBHEt_3_ and subsequent reaction with appropriate dichlorosilane (Method A). Compared to formerly prepared 3, the synthesis of asymmetric compound 4 starts from inexpensive silanes and affords higher yield along with more volatile selenide. Novel four-membered cycle 6 containing two selenium atoms was prepared in the same way in almost quantitative yield, which significantly facilitated its isolation.

Using bulkier *t*Bu_2_SiCl_2_ gave no reaction although the reaction temperature was elevated or BF_3_·OEt_2_ was added. On the contrary, the reaction with Me_2_SiCl_2_ gave tetramethyl-substituted four-membered cycle similarly to 6 (as monitored by GC/MS). However, its very reactive nature made its isolation very difficult even when a strict inert atmosphere was maintained. Tetraethyl derivative 5 showed significantly improved stability and facile isolation but its NMR showed a mixture of two products giving almost the same signals with only small difference in chemical shifts (see the ESI†). On the contrary, no other product was detected by GC/MS. The former preparation of 5 employing Na_2_Se instead of Li_2_Se reported also formation of six-membered cycle containing three Se atoms in 40% yield.^18^ Hence, we prepared Na_2_Se, by reacting Se with NaBHEt_3_, which was subsequently treated with Et_2_SiCl_2_ or *i*Pr_2_SiCl_2_ (Method B) to afford similar mixture of 5 and exclusively 6. Compounds 5 and 6 of the same quality were prepared regardless using Na_2_Se or Li_2_Se and none six-membered cycle was detected in both cases. All of our attempts to isolate and identify the unknown compound prepared along with 5 failed. Since our main goal is to design simple selenium precursor for ALD with easy synthesis and isolation, we have excluded 5 from further studies. When comparing both reactions using Na_2_Se or Li_2_Se (Methods B or A), the latter showed better yields. Employing Method A, tetra*iso*propyl cyclic selenide 6 can be prepared and isolated almost quantitatively.

Selenium can be reduced to silylselenoles or bis(trialkyl)selenides using trialkylsilanes. This was firstly reported for Et_3_Si–SeH (ref. 19) and cHex_3_Si–SeH (ref. 20) and later also for other silanes as demonstrated in our recent work.^21^ Based on these observations, we developed a new synthetic method towards four-membered cycles 6 and 7, which utilizes a direct reaction of elemental selenium with dialkylchlorosilanes in the presence of amine (Method C). Compared to synthesis of former silylselenoles carried out at 250 °C within 48 h, the newly developed method requires only slightly raised reaction temperature (120 °C) and short reaction time (3 h). Moreover, it utilizes readily available and inexpensive starting materials, avoids reactive lithium species and even excludes solvents. Purification of products 6 and 7 is simple and involves only filtration and crystallization at −78 °C. Derivative 6 is stable and can withstand ambient conditions for several hours or even days in case of 7. If stored under an inert atmosphere at −5 °C, no degradation was observed after several months. These properties make 6 and 7 very promising Se-precursors for ALD. A low-yielding (11%) photochemical preparation of 7 including its X-ray structure has been formerly reported by Saak *et al.*^22^ Derivative 6 was prepared for the first time. A similar reaction with Me_2_SiHCl provided a variety of products, which amounts depend on the used temperature and cannot be easily isolated. A small amount of the aforementioned six-membered cycle was detected if the reaction was carried out under elevated temperature to 250 °C (see the ESI† for more details). Structure and purity of the prepared silylselenides have been confirmed by ^1^H/^13^C/^29^Si/^77^Se NMR and GC/MS analysis (see the ESI† for details).

### Thermal properties

**Fig. 1:**
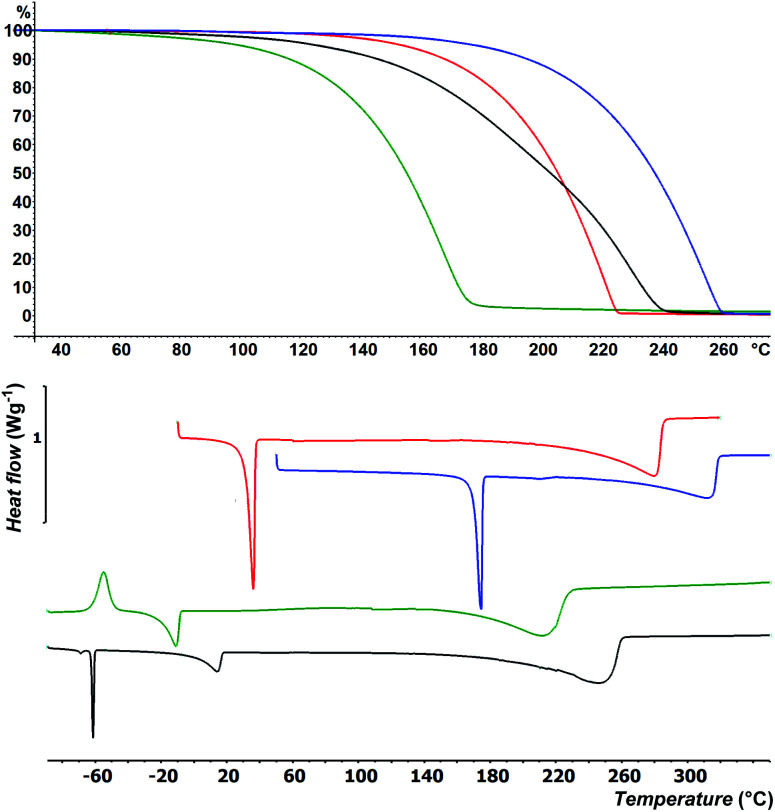
TGA (top) and DSC (bottom) records of 4 (green), 5 (black), 6 (red) and 7 (blue).

Besides easy synthesis and facile isolation, thermal stability and volatility are crucial properties of ALD precursors. Thermal properties of selenides 4–7 were studied by DSC and TG analysis at atmospheric pressure; for thermal properties of 1–3 see our previous communication.^15^ DSC thermograms are shown in Fig. 1 and Table 1 (for complete records including both cooling/heating programs see Fig. S22–S25 in the ESI†). Liquid 4 was firstly cooled to −100 °C, while the sample solidified amorphously at around −90 °C. Its reverse heating revealed cold crystallization at −62 °C followed by broad melting process appearing at −21 °C. The sample began to evaporate at around +150 °C and was completely evaporated at +210 °C. The DSC of liquid 5 showed two peaks of crystallization at 0 and −64 °C under cooling as well as two melting processes (broad at +2 and sharp at −63 °C) under heating program. This is most probably due to aforementioned mixture. Nevertheless, the sample was completely evaporated at +265 °C. Selenide 6 underwent crystallization followed by melting that appeared at +3 and +32 °C. It was evaporated between 220 and 285 °C. Analogical thermal behavior was recorded for 7, which features higher thermal robustness (*T*_m_ = 170 °C, *T*_c_ = 157 °C) and complete evaporation at 315 °C. The TGA shown in Fig. 1 corroborates the DSC measurements and confirmed the good volatility of target organoselenides (see also Fig. S26–S29 in the ESI†). The highest/lowest volatility was observed for derivative 4/7 substituted with methyl/*t*butyl alkyl chains, which implies that thermal properties of 1–7 are easily tunable by proper alkyl substitution. Moreover, zero residues were detected after TGA indicating sufficient thermal stability during heating.

**Table 1:** Fundamental thermal properties of studied cyclic selenides 4–7

Comp.	*T*_c_ a [°C]	*T*_m_ b [°C]	*T*_E_ c [°C]
4	−62	−21	+150 to +210
5	−64/0	−63/+2	+160 to +265
6	+3	+32	+220 to +285
7	+157	+170	+250 to +315

a Temperature of crystallization.

b Temperature of melting.

c Range of evaporation.

### Preparation of MoSe_2_ flakes by ALD

**Fig. 2:**
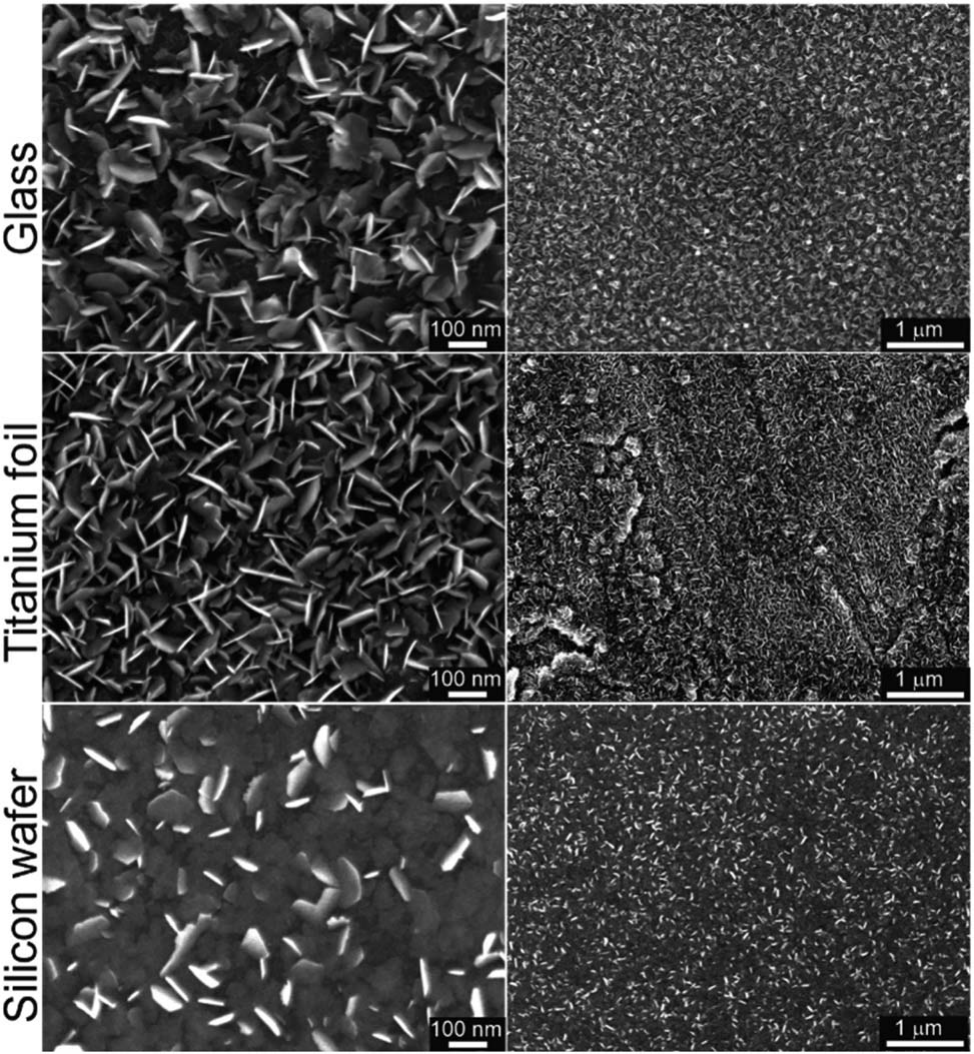
SEM top images at two different magnifications of the as-deposited ALD MoSe_2_ at 300 °C upon 800 cycles (800 ms Se dose) on different substrates. The as-deposited MoSe_2_ shows 2D flaky nanosheets morphology mainly out-of-plane oriented.

In our recent work on MoSe_2_ deposition by ALD,^15^ we have identified that six-membered silylselenides bearing two selenium atoms performed better than five-membered analogues. Especially compound 3 showed outstanding performance in MoSe_2_ deposition. Hence, we have selected selenide 6 as a model compound for surface reaction with MoCl_5_ and deposition of MoSe_2_. It features four-membered structure bearing two selenium atoms and facile preparation, easy isolation, good volatility and the highest attained yields using Methods A–C. The deposition of MoSe_2_ has been carried out in a custom thermal ALD system (see Experimental part for ALD process details). Glass, annealed titanium foil (with TiO_2_ surface in the anatase phase) and silicon wafer (with SiO_2_ surface) were used as substrates. The ALD cycle was comprised of four steps described as follows: Se precursor (800 ms)–N_2_ purge (5 s)–Mo precursor (800 ms)–N_2_ purge (5 s). The number of cycles applied were 800 and the deposition temperature was 300 °C (detailed description in Experimental section). The morphology and structure of the as-deposited MoSe_2_ on the different substrates was characterized by means of scanning electron microscope (SEM) as displayed in Fig. 2. Therein, one can observe the MoSe_2_ grew as 2D flaky nanosheets, mainly oriented out-of-plane.

ALD processes applying same number of cycles but different Se dose, namely 400 and 1200 ms (while keeping Mo dose 800 ms), were conducted in order to verify the self-limiting nature of the process. Corresponding SEM top view images from annealed Ti foils and Si wafer, together with the SEM cross-section of the Si wafer, are shown in the Fig. S30.† Therein, the MoSe_2_ deposited applying a Se dose of 800 and 1200 ms showed similar features, *i.e.* the density and size of MoSe_2_ flakes, indicating a saturation regime and confirming the self-limiting nature of the process.

Additionally, the deposition temperature dependence was studied conducting ALD processes (800 cycles) at different temperatures, 250 and 200 °C (in addition to 300 °C). In contrast to 2D flaky crystallites obtained at 250 °C, the as-deposited MoSe_2_ at 200 °C exhibited granular morphology (see Fig. S31† for the corresponding SEM images). These results revealed a thermal dependence of as-deposited MoSe_2_ morphology.

**Fig. 3:**
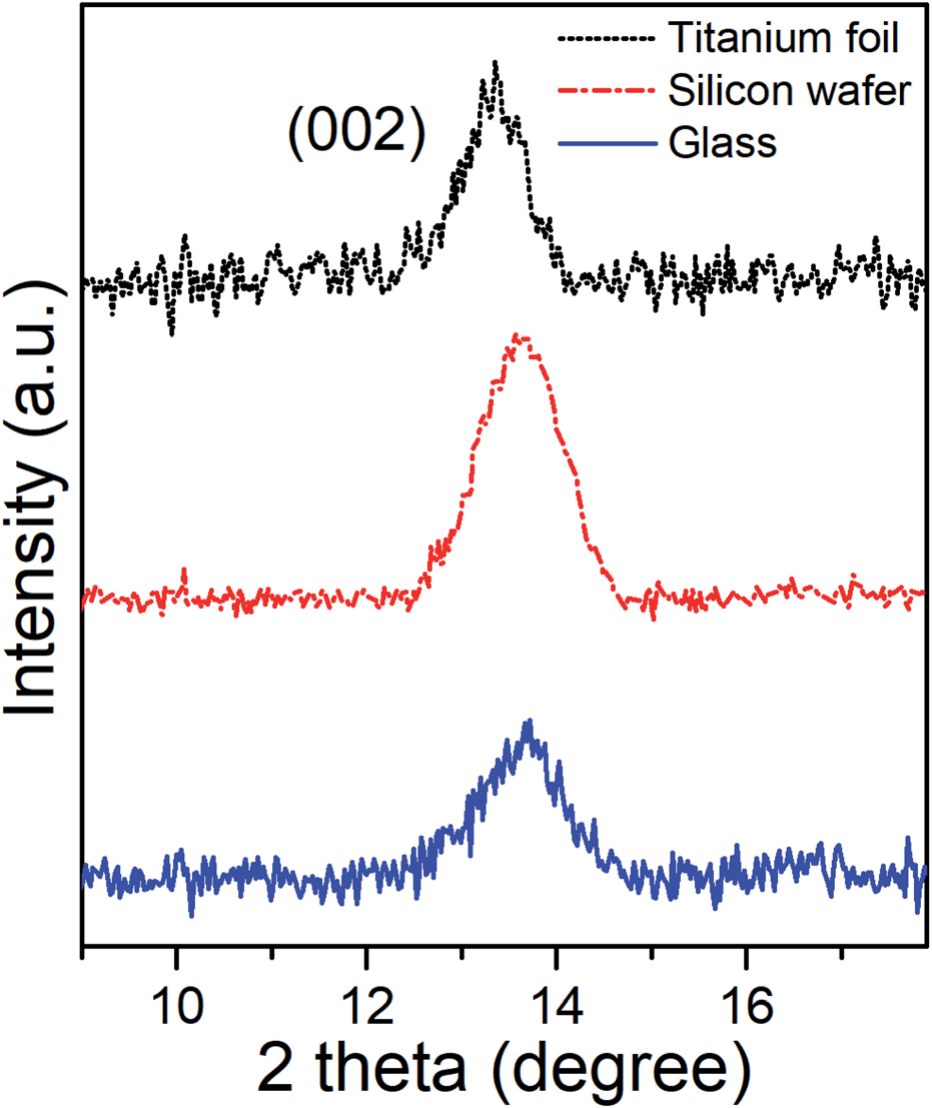
XRD patterns of the as-deposited ALD MoSe_2_ on different substrates upon 800 ALD cycles (800 ms Se dose). The plane (002) revealed the out-of-plane orientation of the as-deposited ALD MoSe_2_ at 300 °C onTianium foil (top), silicon wafer (middle) and glass (down).

Grazing Incident X-Ray Diffractometry (GI-XRD) characterization of as-deposited MoSe_2_ at 300 °C provided the corresponding GI-XRD patterns on the different substrates shown in the Fig. 3. The patterns clearly exhibited diffraction peaks at 2*θ* ∼ 13.5°, which matched well with the (002) plane of hexagonal (2H) MoSe_2_, and confirmed the presence of MoSe_2_ with out-of-plane orientation in line with the SEM images.

**Fig. 4:**
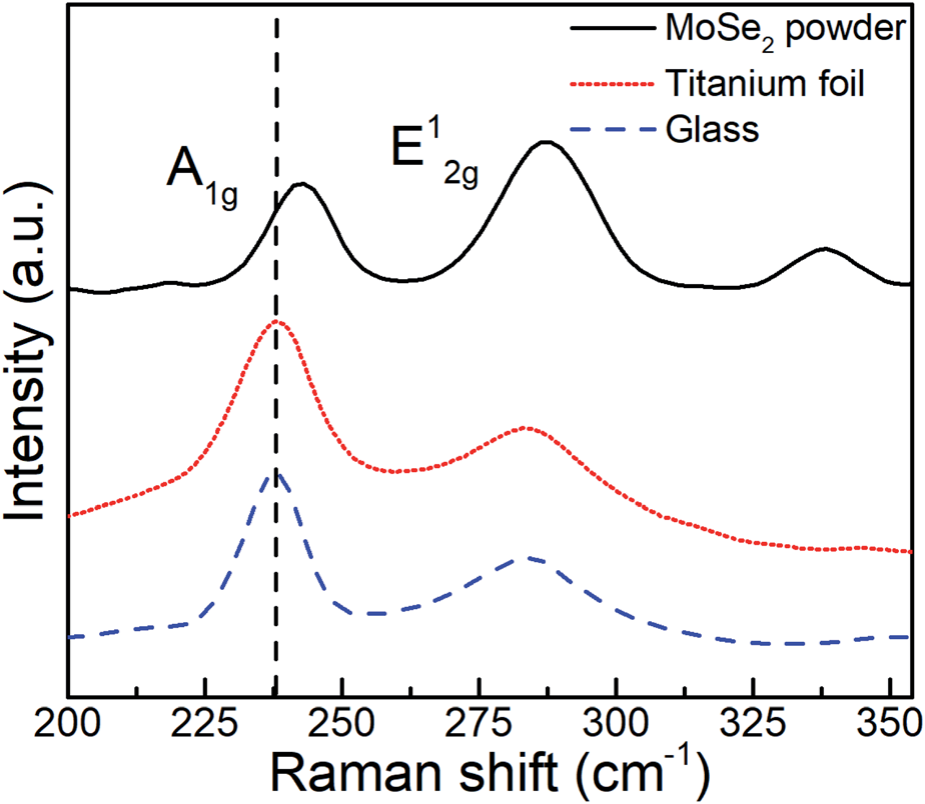
Raman spectra obtained from (top) MoSe_2_ powder and as-deposited ALD MoSe_2_ on titanium foil (middle) and glass (down) at 300 °C upon 800 ALD cycles (800 ms Se dose).

The as-deposited MoSe_2_ structure was further characterized by means of Raman spectroscopy, a paramount technique for the assessment of layered materials. The corresponding Raman spectra and obtained from as-deposited MoSe_2_ at 300 °C (see Fig. 4) and 200 and 250 °C (see Fig. S32†) exhibited characteristic 2H-MoSe_2_ peaks, namely, A_1g_ (out-of-plane) and E^1^_2g_ (in-plane) modes at ∼238 and ∼285 cm^−1^, respectively.^23^

The few-layered nature of the deposited 2D MoSe_2_ was confirmed by the red shift of the A_1g_ peak as compared to the peak position for MoSe_2_ powder (242 cm^−1^), while the observed difference in the relative intensities between the A_1g_ and E^1^_2g_ modes indicated the prevailing out-of-plane orientation, as observed in the SEM images and XRD results.^24^

**Fig. 5:**
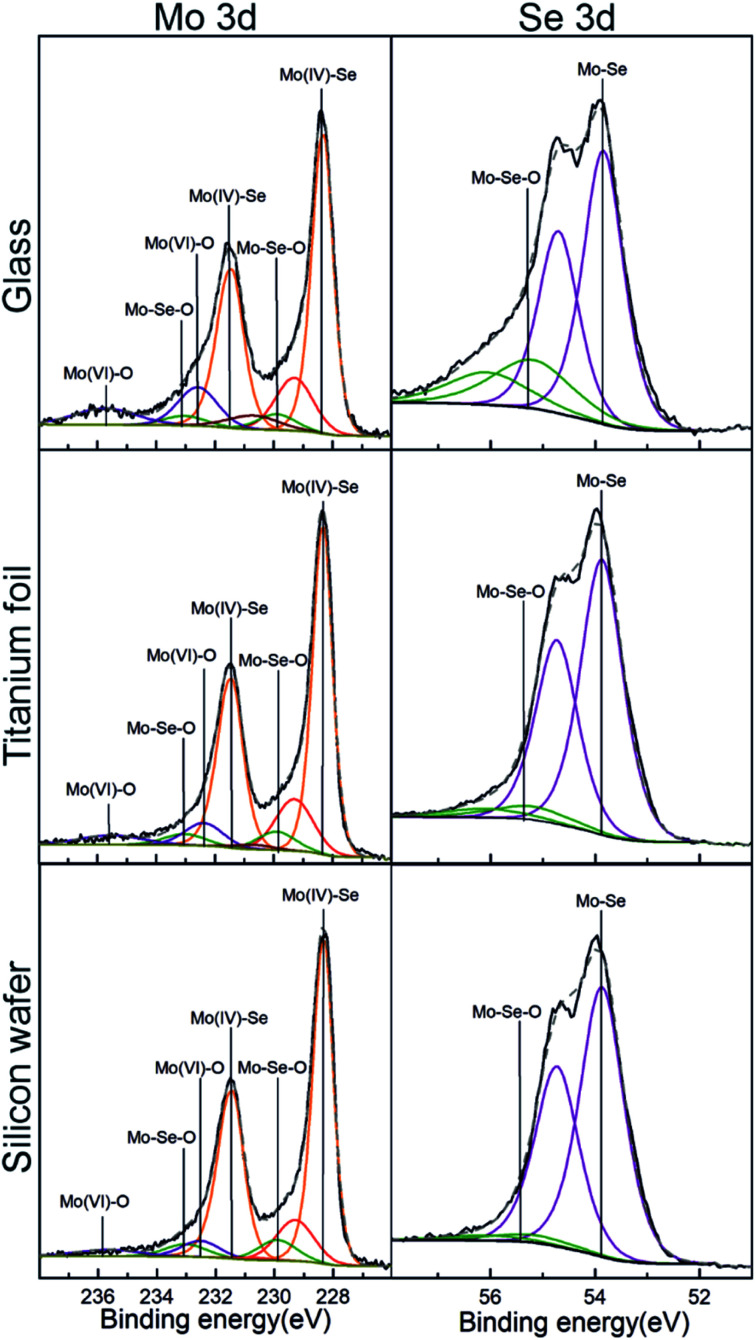
XPS high-resolution spectra of Mo 3d (left) and Se 3d (right) corresponding to as-deposited ALD MoSe_2_ upon 800 ALD cycles at 300 °C (800 ms Se dose).

X-ray Photoelectron Spectroscopy (XPS) analysis was conducted to assess the surface chemical composition of the as-deposited MoSe_2_. Here, it is important to note that the use of adventitious carbon (284.8 eV) as a reference to adjust the binding energy scale was not reliable, basically due to the strong overlapping of C 1s signal with Se LMM. Instead, the binding energy of Mo 3d at 228.3 eV corresponding to MoSe_2_ was used for this purpose.^25,26^ Fig. S33† shows the XPS survey spectra for MoSe_2_ deposited at 300 °C on glass, annealed Ti foils and Si wafer, whereas Fig. S34† exhibits the XPS survey spectra obtained for MoSe_2_ deposited at 200 and 250 °C on Si wafer. The left column in Fig. 5, shows the deconvolution of the XPS high resolution spectra of Mo 3d obtained from as-deposited ALD MoSe_2_ at 300 °C on the different substrates. The most intense doublets (orange) centered at 228.3 and 231.4 eV corresponds to Mo(iv)–Se, corroborating the growth of MoSe_2_. The peaks at 229.9 and 233.0 eV (green) were attributed to Mo–Se–O. Actually, the presence of Mo–Se–O bonds evidences both the chemical reaction of the Se precursor with the hydroxyl groups from the substrates surface, and the posterior reaction of the resulting chemisorbed counterpart with the Mo precursor. The last doublet at ∼232.5 and 235.7 eV (blue) was associated with Mo(vi)–O, originated upon the reaction of MoCl_5_ with the hydroxyl groups from the substrate surface during the early stage of the deposition process. Regarding the deconvolution of the XPS high resolution spectra of Se 3d (right column of Fig. 5), it exhibited four components ascribed to two Se 3d_5/2_ and Se 3d_3/2_ spin–orbit splitting. The first and most intense doublet located at 53.8 and 54.7 eV, corresponding to Mo–Se bonds, which unambiguously corroborated the growth of MoSe_2_. And the second doublet with the peaks centered at 55.2 and 56.1 eV, attributed to Mo–Se–O, confirming the presence of this species in Mo 3d. Regarding the MoSe_2_ deposited at 250 °C (see Fig. S35†) the deconvoluted XPS high resolution spectra of Mo 3d and Se 3d exhibited the same features than those described for MoSe_2_ deposited at 300 °C. In contrast, the MoSe_2_ deposited at 200 °C (Fig. S35†) displayed a doublet at 54.7 and 55.6 eV in the Se 3d deconvoluted XPS high resolution spectra ascribed to Se–Se or Se^0^. This would suggest that the chemical reaction between the Mo and Se precursors could be thermally limited at a deposition temperature of 200 °C.

As compared to the XPS results obtained in our previous work from the MoSe_2_ deposited using selenides 1 and 3,^15^ one must notice relevant differences: (i) the absence of chlorine residues (mainly present by Mo–Cl species) and (ii) the well-defined Se 3d doublet. Those differences suggested the absence of impurities (other chemical species) and complete ligand exchange reaction between selenide 6 and MoCl_5_ resulting in a high purity as-deposited ALD MoSe_2_.

## Conclusions

In summary, we have investigated a series of four-, five- and six-membered cyclic silylselenides as potential Se-precursors for ALD. They were prepared in a straightforward manner employing elemental selenium and chlorosilanes. The solvent-free Method C, which starts from Se and commercially available and inexpensive chlorosilanes, is in particular operationally very simple, avoids generation of lithium or sodium selenides and affords four-membered derivatives 6 and 7 in good yields of 77 and 61%, respectively. The performed DSC and TG analysis revealed that volatility can be tailored by choosing proper peripheral alkyl substituents. Especially novel silylselenide 6 bearing *iso*propyl groups showed very good trade-off between its chemical stability and sufficient volatility. Its ALD reaction with MoCl_5_ successfully provided 2D MoSe_2_ nanosheets that were characterized by SEM, GI-XRD, Raman spectroscopy and XPS. Compared to previous silylselenides, precursor 6 showed complete ligand exchange reaction and its ALD afforded MoSe_2_ of high purity.

## Experimental

The NMR and GC/MS spectra were recorded with a Bruker AVANCE 400 instrument and a GC/EI-MS configuration including gas chromatography Agilent Technologies 6890N (HP-5MS, 30 m column, I.D. 0.25 mm, film 0.25 µm) equipped with a mass detector Network MS detector 5973 (EI 70 eV, range 33–550 Da). Me_4_Si and Me_2_Se were used as internal standards for ^1^H/^13^C/^29^Si and ^77^Se NMR measurements (*δ* = 0 ppm). Thermal properties of target molecules were measured by differential scanning calorimetry (DSC) with a Mettler-Toledo STARe System DSC 2/700 equipped with FRS 6 ceramic sensor and cooling system HUBER TC100-MT RC 23 or by thermogravimetric analysis (TGA) with a Mettler-Toledo STARe System TGA 2 equipped with a horizontal furnace LF (400 W, 1100 °C), balance XP5 (resolution 1 µg) and cooling system HUBER Minichiller 600. DSC thermograms of the target compounds were measured in aluminous crucibles with a small hole in the lid under N_2_ inert atmosphere. DSC curves were determined with a scan rate of 5 °C min^−1^ within the range −100 °C to +400 °C.

The synthesis and workup were performed under Ar atmosphere or in a nitrogen-filled glovebox. All used solvents were properly dried before use. The used glassware was poured into sodium hypochlorite bath to destroy remaining organic selenides before cleaning.

### General method A

Dry THF (40 ml) and fine selenium powder (1.0 g, 12.6 mmol) were placed to a 100 ml Schlenk flask. The suspension was cooled to 0 °C and LiBHEt_3_ (25.2 ml, 25.2 mmol, 1 M solution in THF) was added slowly. The mixture was stirred at 25 °C for 2 h forming white suspension of Li_2_Se. The corresponding dialkyldichlorosilane (1 or 0.5 eq. for 5/6 or 4) dissolved in THF (10 ml) was added dropwise and the reaction mixture was stirred at 25 °C for 15 h. The solvent was evaporated *in vacuo*, dry hexane (30 ml) was added and the solution was filtered *via* a cannula. The hexane solutions of 4 or 5 were evaporated *in vacuo* and the remaining yellowish oil was purified by vacuum distillation. The hexane solution of 6 was cooled to −78 °C to afford colourless solid, while the supernatant was removed *via* a syringe. The final product was dried *in vacuo*.

### General method B

Dry THF (40 ml) and fine selenium powder (1.0 g, 12.6 mmol) were placed to a 100 ml Schlenk flask. The suspension was cooled to 0 °C and NaBHEt_3_ (25.2 ml, 25.2 mmol, 1 M solution in THF) was added slowly. The mixture was stirred at 25 °C for 2 h forming dark violet suspension of Na_2_Se. The remaining procedure is similar to Method A.

### General method C

Elemental selenium (1.0 g, 12.6 mmol) along with dialkylchlorosilane (1 eq.) and EtNiPr_2_ (2.2 ml, 1.64 g, 12.6 mmol) were introduced into a pressure vessel filled with argon and containing a magnetic stirrer. The mixture was stirred at 120 °C for 3 h and then was cooled to 25 °C to solidify. A second stirrer was added along with hexane (15 ml) and the mixture was vigorously stirred and shook until a yellow solution with a white precipitate were formed. The mixture was filtered *via* cannula and cooled to −78 °C to afford yellowish crystals, while the hexane was removed *via* a syringe. The crystal product was dried *in vacuo*.

### 2,2,4,4,5,5-Hexamethyl-1,3,2,4,5-diselenatrisilolane (4)

The title compound was prepared from Me_4_Si_2_Cl_2_ (1.2 ml, 1.2 g, 6.3 mmol) and Me_2_SiCl_2_ (0.8 ml, 0.8 g, 6.3 mmol) following the Method A. Yellowish liquid (1.6 g, 75%) with bp = 95–100 °C (1 torr). ^1^H-NMR (400 MHz, 25 °C, C_6_D_6_): *δ*(^1^H) = 0.45 (s, 12H, CH_3_), 0.78 (s, 6H, CH_3_) ppm. ^13^C-NMR APT (100 MHz, 25 °C, C_6_D_6_): *δ*(^13^C) = 0.6, 9.9 ppm. ^29^Si-NMR (80 MHz, 25 °C, C_6_D_6_): *δ*(^29^Si) = 13.9, 24.9 ppm. ^77^Se-NMR (76 MHz, 25 °C, C_6_D_6_): *δ*(^77^Se) = −303.5 ppm. EI-MS: *m*/*z* = 319 (30), 211 (20), 73 (100).

### 2,2,4,4-Tetraethyl-1,3,2,4-diselenadisiletane (5)

The title compound was prepared from Et_2_SiCl_2_ (1.9 ml, 1.99 g, 12.6 mmol) following the Method A (1.9 g, 92%) or Method B (0.9 g, 43%). Compound 5 was prepared as a mixture with inseparable impurity. Yellow liquid with bp = 120–140 °C (1 torr). EI-MS: *m*/*z* = 332 (20, M^+^), 303 (100), 275 (30), 245 (20), 217 (20), 59 (10). For the NMR spectra see the ESI.†

### 2,2,4,4-Tetra*iso*propyl-1,3,2,4-diselenadisiletane (6)

The title compound was prepared from *i*Pr_2_SiCl_2_ (2.3 ml, 2.3 g, 12.6 mmol) following the Method A (2.4 g 99%) or Method B (1.8 g, 72%). It was also prepared from *i*Pr_2_SiHCl (2.2 ml, 1.9 g, 12.6 mmol) following the Method C (1.9 g, 81%). Yellowish liquid. ^1^H-NMR (400 MHz, 25 °C, C_6_D_6_): *δ*(^1^H) = 1.15 (d, *J* = 7 Hz, 24H, CH_3_), 1.21–1.30 (m, 4H, CH) ppm. ^13^C-NMR APT (100 MHz, 25 °C, C_6_D_6_): *δ*(^13^C) = 17.72, 18.37 ppm. ^29^Si-NMR (80 MHz, 25 °C, C_6_D_6_): *δ*(^29^Si) = 18.1 ppm. ^77^Se-NMR (76 MHz, 25 °C, C_6_D_6_): *δ*(^77^Se) = −402.33 ppm. EI-MS: *m*/*z* = 388 (10, M^+^), 345 (100), 303 (20), 275 (20), 231 (20), 59 (10).

### 2,2,4,4-Tetra-*tert*-butyl-1,3,2,4-diselenadisiletane (7)

The title compound was prepared from*t*Bu_2_SiHCl (2.6 ml, 2.3 g, 12.6 mmol) following the Method C (1.7 g, 61%). Orange crystals. ^1^H-NMR (400 MHz, 25 °C, C_6_D_6_): *δ*(^1^H) = 1.23 (s, 36H, CH_3_) ppm. ^13^C-NMR APT (100 MHz, 25 °C, C_6_D_6_): *δ*(^13^C) = 25.12, 29.19 ppm. ^29^Si-NMR (80 MHz, 25 °C, C_6_D_6_): *δ*(^29^Si) = 19 ppm. ^77^Se-NMR (76 MHz, 25 °C, C_6_D_6_): *δ*(^77^Se) = −321.9 ppm. EI-MS: *m*/*z* = 387 (100), 345 (80), 246 (20), 57 (20).

### Deposition of MoSe_2_

The deposition of MoSe_2_ was carried out in a custom-made thermal ALD system applying a deposition temperature of 300 °C at a chamber pressure of 2 mbar. The Mo precursor, MoCl_5_ (Strem, anhydrous 99.6%), and synthesized 6 were heated up to get sufficiently high vapour pressure at 120 and 155 °C, respectively. The precursors-enriched carrier gas was delivered through separate heated stainless steel lines (separate for each precursor) directly into the cylindrical deposition chamber of diameter 50 mm and length 300 mm. The substrates were placed on the stainless steel holder of dimensions 35 × 80 mm placed in the centre of the precisely temperature-controlled chamber. The MoSe_2_ ALD process started immediately after 5 pulses of ultrapure water, applied to increase the number of hydroxyl active sites on the substrates surface. The ALD cycle was comprised of four steps described as follows: Se precursor (800 ms)–N_2_ purge (5 s)–Mo precursor (800 ms)–N_2_ purge (5 s). The number of cycles applied was 800. In parallel, ALD processes applying the same number of cycles but different Se dose, namely 400 and 1200 ms (for a fixed Mo dose of 800 ms) were conducted in order to verify the self-limiting nature of process. As to the deposition temperature dependence, it was evaluated by ALD processes conducted at 200 and 250 °C (in addition to 300 °C). N_2_ (99.999%) was used as a carrier gas. The precursors were boosted from the heated standard stainless steel bubblers (Strem, catalog no. 98-0276) at a flow rate of 40 standard cubic centimeter per minute (sccm) in all processes.

### MoSe_2_ characterization

The structure and morphology of the as-deposited MoSe_2_ were assessed by field emission scanning electron microscope (FE-SEM JEOL JSM 7500F).

X-ray diffraction (XRD) analysis was carried out using Panalytical Empyrean with Cu tube and Pixcel3D detector. Grazing incidence XRD was performed applying an incident angle of 1 degree. The patterns were recorded in range of 5°–65°, step size was 0.026 degree.

Raman micro-spectrometer HORIBA LabRAM HR Evolution system coupled by with a confocal microscope was to conduct Raman measurements taken by 532 nm (green) laser excitation source in the range 100–500 cm^−1^. All spectra were carefully corrected by baseline correction and noise reduction. Spikes were eliminated by spectra accumulation or manually in the LabSpec 6 software.

The surface chemical composition of MoSe_2_ was monitored by X-ray photoelectron spectroscopy (XPS) (ESCA2SR, Scienta-Omicron) using a monochromatic Al Kα (1486.7 eV) X-ray source. Due to the strong overlapping of C 1s signal with Se LMM, the binding energy scale was referenced to the binding energy of Mo 3d at 228.3 eV corresponding to MoSe_2_.^25,26^ The deconvolution of the Mo 3d spectra included the use of eight components since Mo 3d has a strong overlapping with the Se 3s signal. From those, six components corresponded to the three spin–orbit splitting of Mo 3d, Mo 3d_5/2_ and Mo 3d_3/2_, *i.e.* three chemical species and the remaining two correspond to Se 3s signals.

## Conflicts of interest

There are no conflicts to declare.

## Author contributions

Funding acquisition: FB, JMM; investigation: JC, RZ, RK, JR-P, DP, DP, MK, VJ; methodology: JC, RZ; project administration: FB, JMM; writing – original draft: JC, RZ; writing – review & editing: FB.

## Supplementary Material

RA-011-D0RA10239C-s001
