# Supplementary material for: Deposition of MoSe2 flakes using cyclic selenides
Source: RSC Adv. 2021 Jun 23;11(36):22140–7. doi: 10.1039/d0ra10239c (PMC9034216; doi:10.1039/d0ra10239c)
Supplement: RA-011-D0RA10239C-s001 [file RA-011-D0RA10239C-s001.pdf]

Electronic Supplementary Material (ESI) for RSC Advances.
This journal is © The Royal Society of Chemistry 2021

Electronic Supporting Information

Deposition of $MoSe_2$ flakes using cyclic selenides

Jaroslav Charvot,[a] Raul Zazpe,[b,c] Richard Krumpolec,[d] Jhonatan Rodriguez-Pereira,[b,c] David Pavliňák,[d] Daniel Pokorný,[a] Milan Klikar,[a] Veronika Jelínková,[e] Jan M. Macak [b,c] and Filip Bureš*[a,e]

[a]Institute of Organic Chemistry and Technology, Faculty of Chemical Technology, University of Pardubice, Studentská 573, Pardubice, 53210, Czech Republic.

[b]Center of Materials and Nanotechnologies, Faculty of Chemical Technology, University of Pardubice, Nám. Čs. Legií 565, Pardubice, 53002, Czech Republic.

[c]Central European Institute of Technology, Brno University of Technology, Purkyňova 123, Brno, 61200, Czech Republic.

[d]Department of Physical Electronics, CEPLANT — R&D Center for Plasma and Nanotechnology Surface Modifications, Faculty of Science, Masaryk University, Kotlářská 267/2, 61137 Brno, Czech Republic

[e]The Institute of Technology and Business in České Budějovice, Okružní 517/10, 370 01, České Budějovice, Czech Republic

Table of contents

1. Synthesis 2
   1.2. Attempted preparation of 2,2,4,4-tetramethyl-1,3,2,4-diselenadisiletane 2
2. Nuclear magnetic resonance spectroscopy 3
   2.1. ^{1}H NMR spectra 3
   2.2. ^{13}C NMR spectra 5
   2.3. ^{29}Si NMR spectra 7
   2.4. ^{77}Se NMR spectra 9
3. GC/MS records 11
4. DSC thermograms 15
5. TGA 17
6. SEM 19
7. Raman spectroscopy 20
8. XPS 21

1. Synthesis

1.2. Attempted preparation of 2,2,4,4-tetramethyl-1,3,2,4-diselenadisiletane

The title compound was attempted from Me_2SiCl_2 (1.6 ml, 1.6 g, 12.6 mmol) following the Methods A or B as well as from MeSiHCl (1.4 ml, 1.2 g, 12.6 mmol) following the Method C. The crude product is a yellow oil. All three methods provided similar results. Figure S1 shows GC/MS records of the crude reaction mixture using Method C at 100 and 250 °C. Three main products **A**–**C** were identified but the reproducibility of these experiments was rather low. The title compound (**A**) was identified in the crude reaction mixture but all attempts on its purification, including vacuum distillation, crystallization at –78 °C and sublimation, failed.

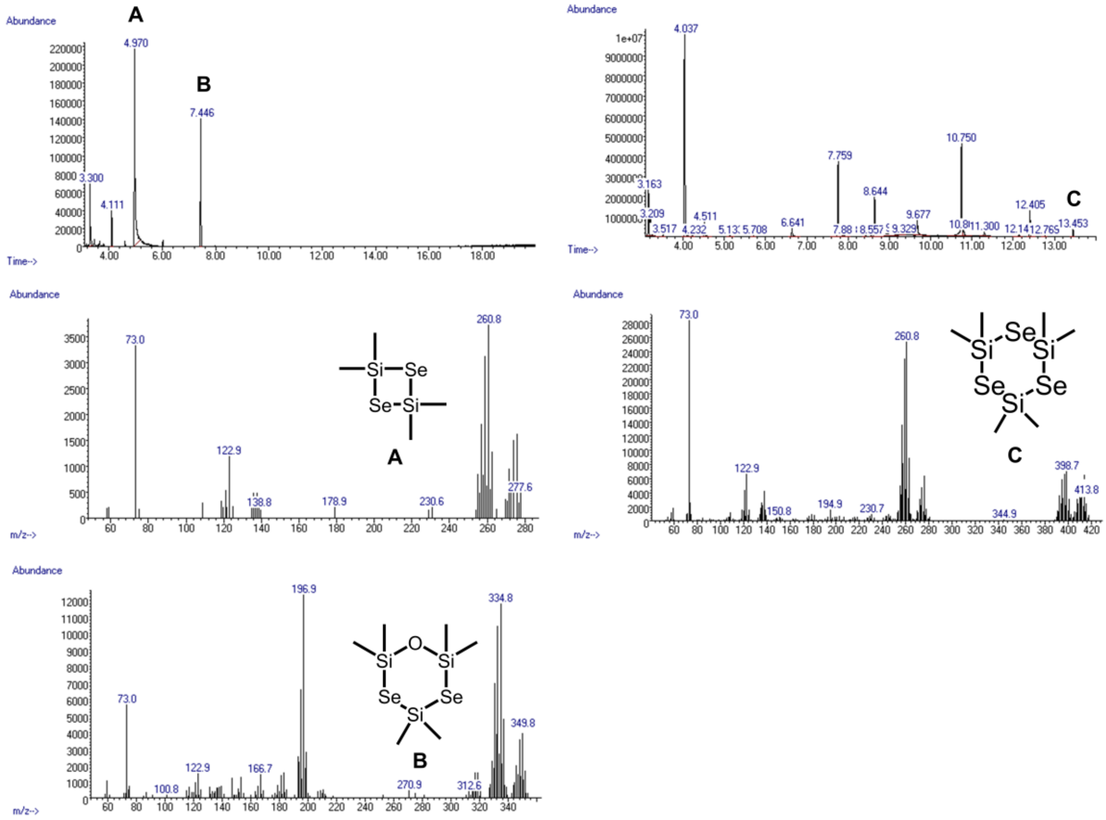

Figure S1 GC/MS record of crude reaction mixture during attempted preparation of 2,2,4,4-tetramethyl-1,3,2,4-diselenadisiletane. Method C at 100 (left) and 250 °C (right).

2. Nuclear magnetic resonance spectroscopy

2.1. ^{1}H NMR spectra

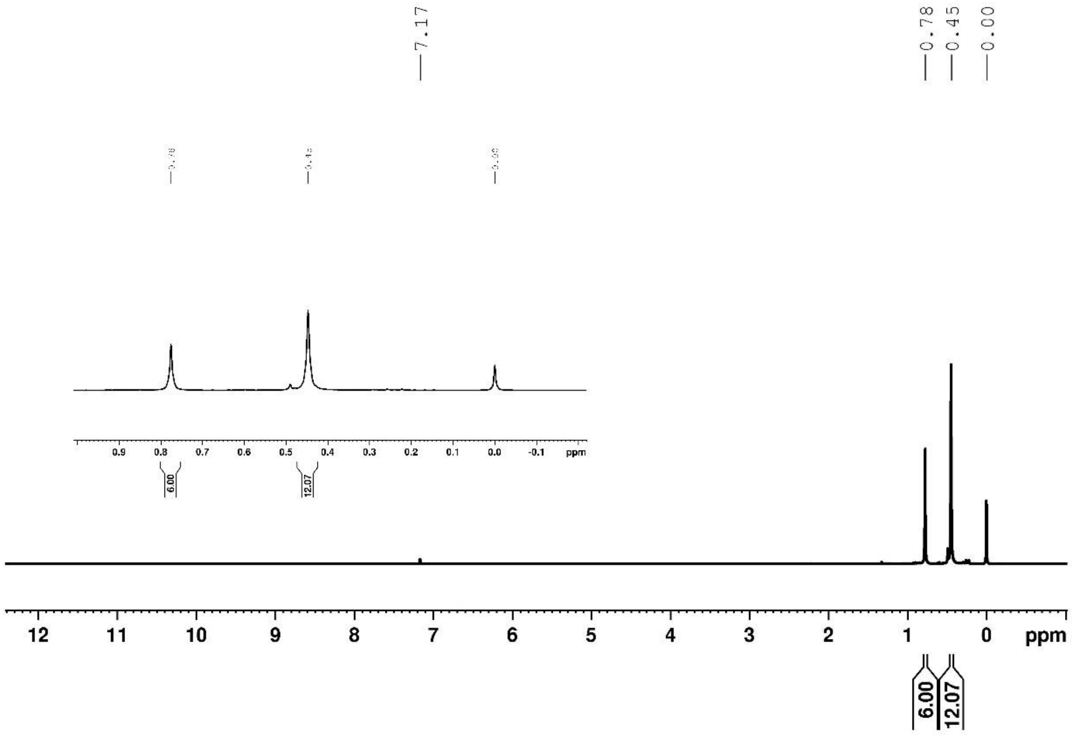

Figure S2 ^{1}H-NMR (400 MHz, 25 °C, C_6D_6) spectra of **4**.

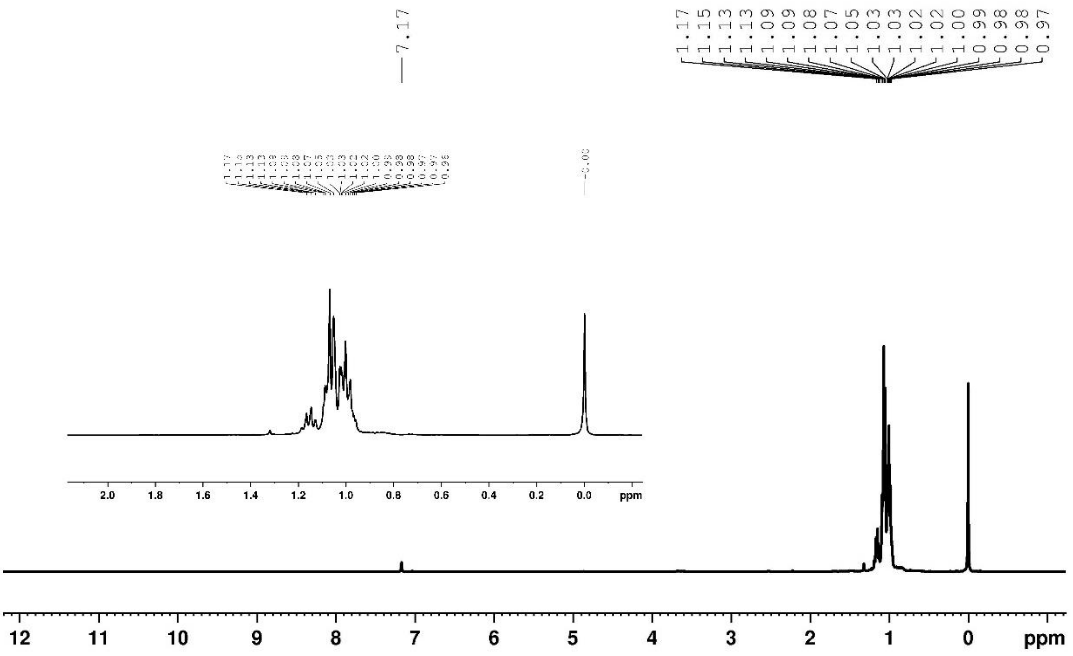

Figure S3 ^{1}H-NMR (400 MHz, 25 °C, C_6D_6) spectra of **5** (mixture).

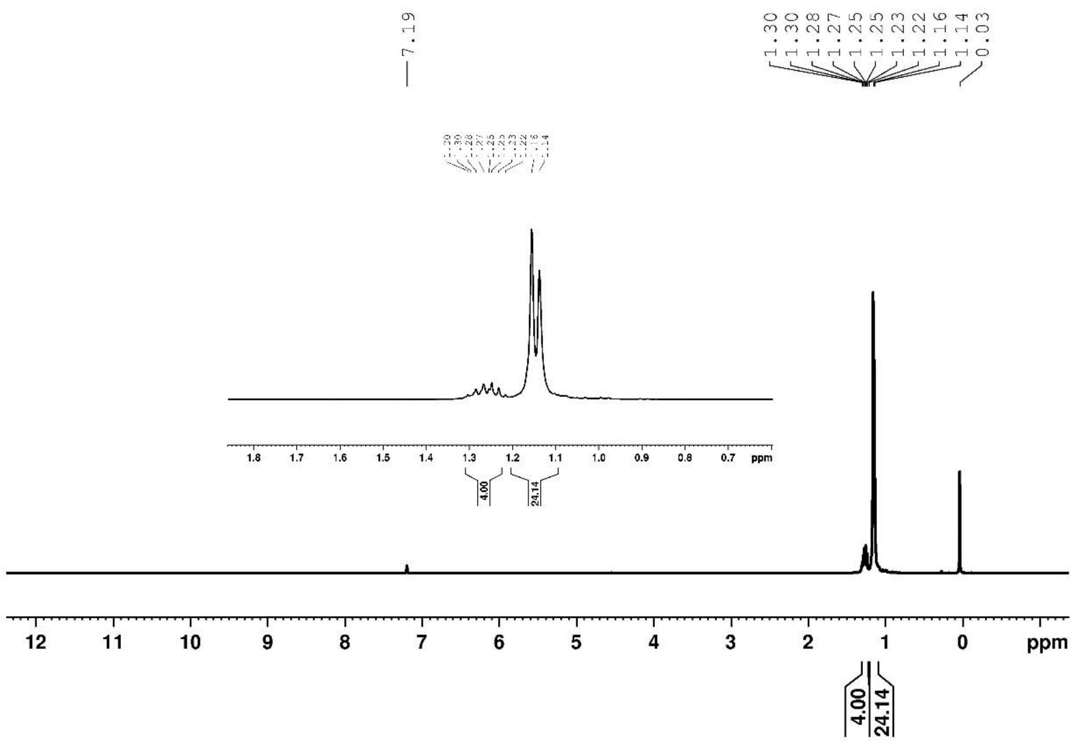

Figure S4 ^{1}H-NMR (400 MHz, 25 °C, C_6D_6) spectra of **6**.

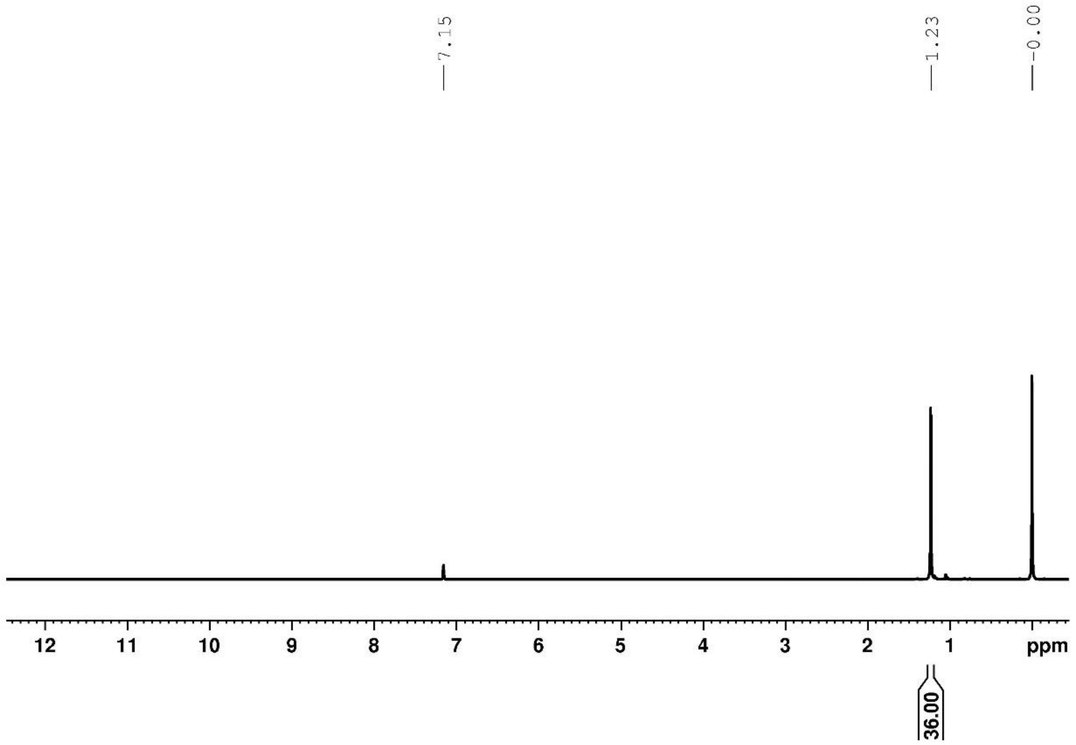

Figure S5 ^{1}H-NMR (400 MHz, 25 °C, C_6D_6) spectra of **7**.

2.2. ^{13}C NMR spectra

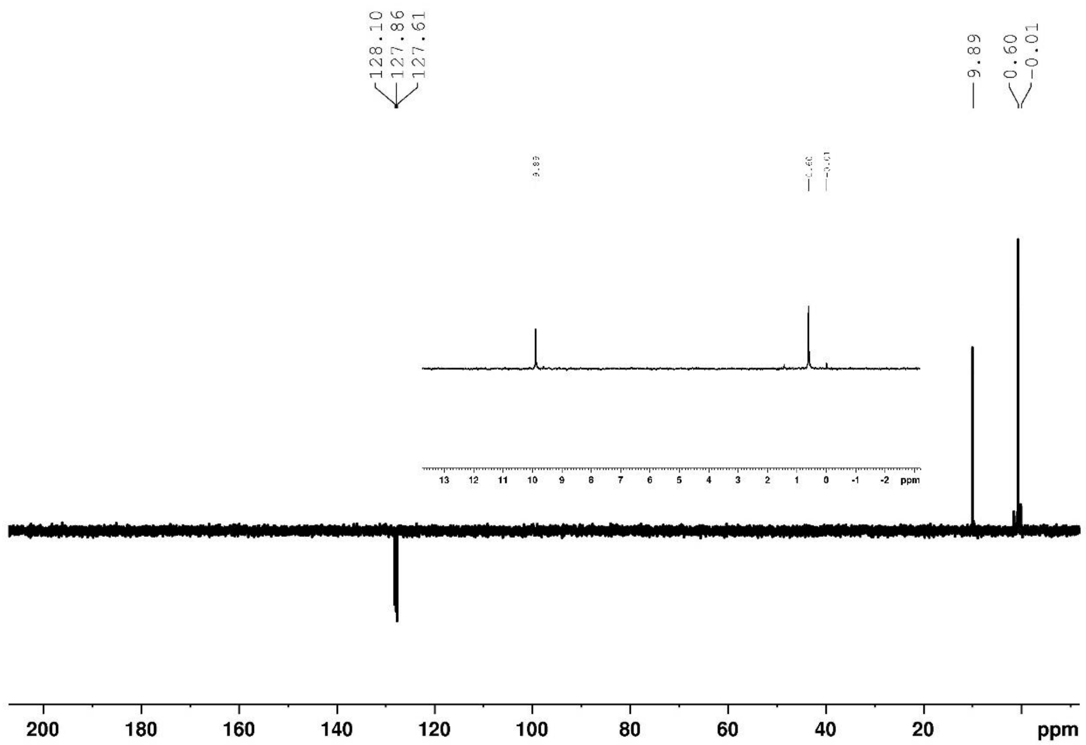

Figure S6 ^{13}C-NMR APT (100 MHz, 25 °C, C_6D_6) spectra of **4**.

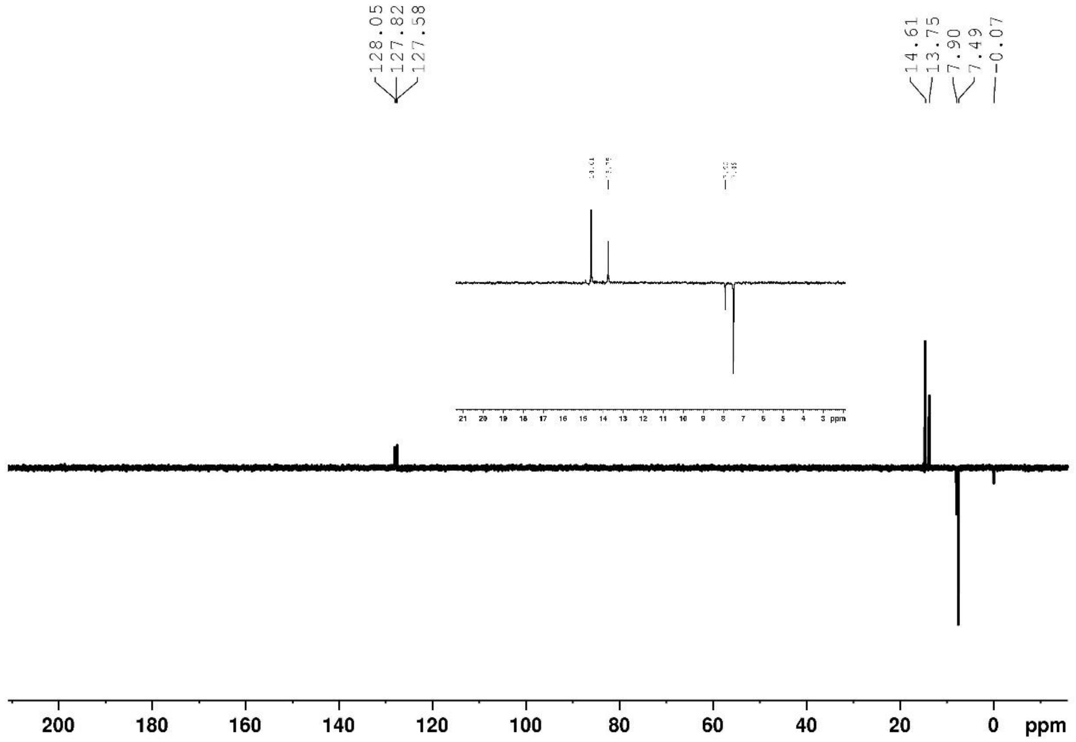

Figure S7 ^{13}C-NMR APT (100 MHz, 25 °C, C_6D_6) spectra of **5** (mixture).

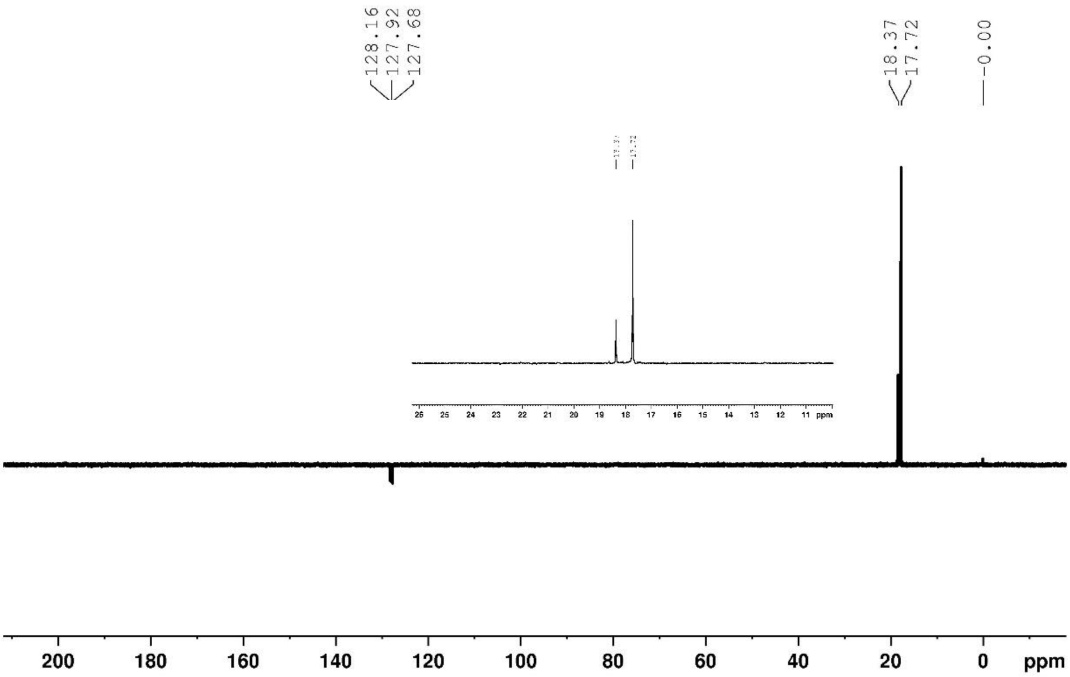

Figure S8 ^{13}C-NMR APT (100 MHz, 25 °C, C_6D_6) spectra of **6**.

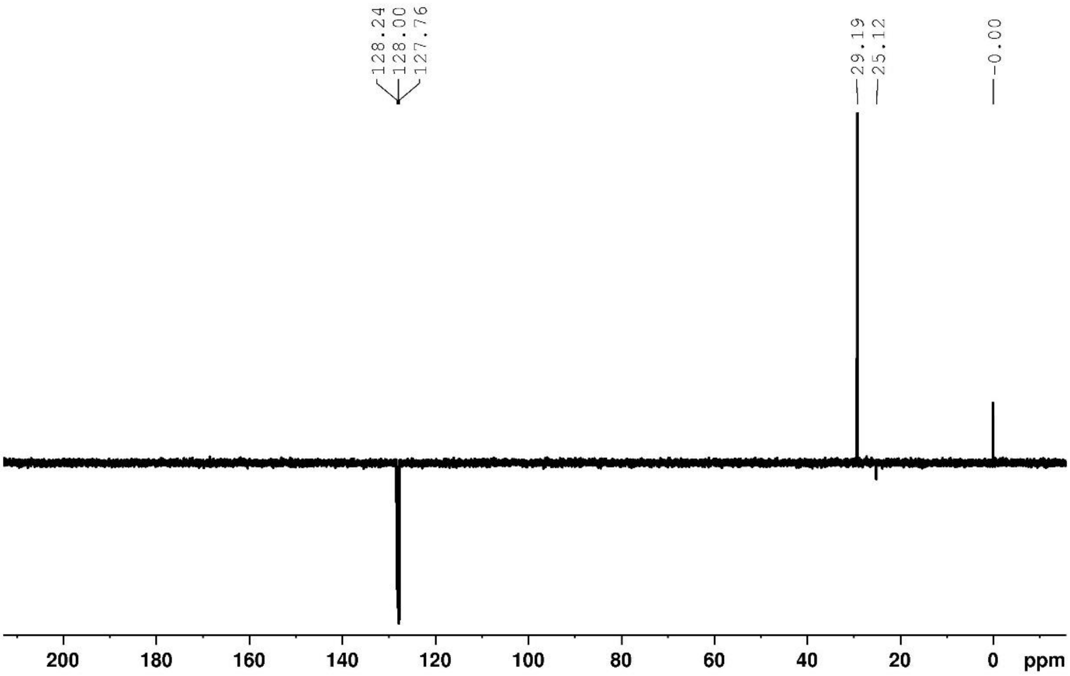

Figure S9 ^{13}C-NMR APT (100 MHz, 25 °C, C_6D_6) spectra of **7**.

^{1}H-NMR (400 MHz, 25 °C, C_6D_6) spectra of **7**.

2.3. ^{29}Si NMR spectra

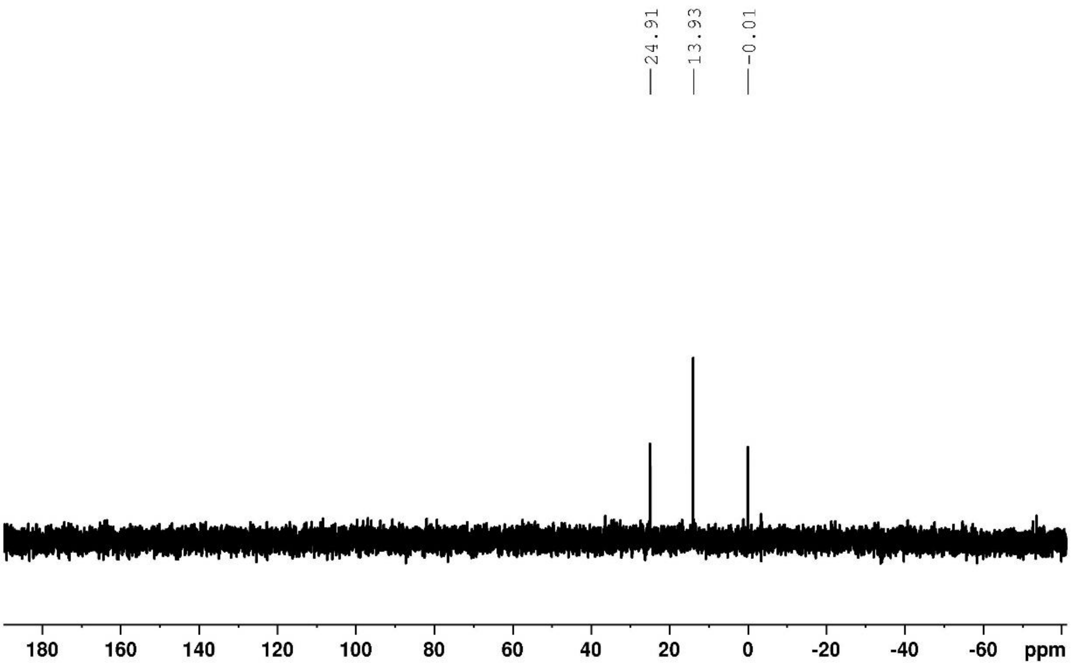

Figure S10 ^{29}Si-NMR (80 MHz, 25 °C, C_6D_6) spectra of **4**.

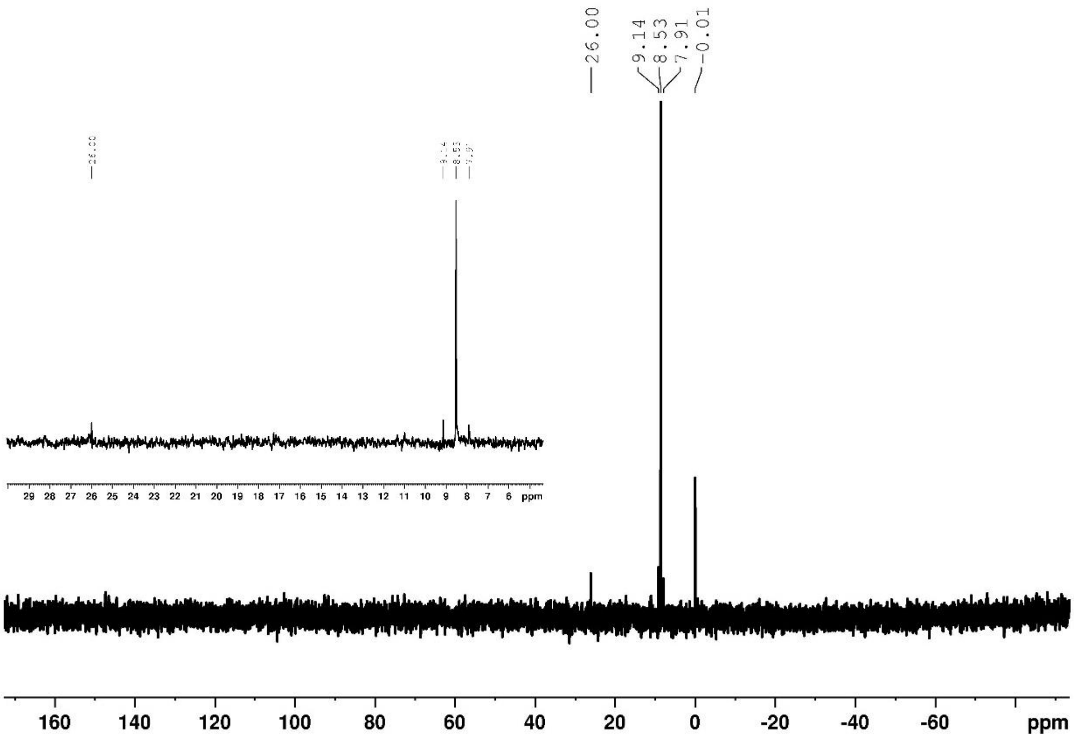

Figure S11 ^{29}Si-NMR (80 MHz, 25 °C, C_6D_6) spectra of **5** (mixture).

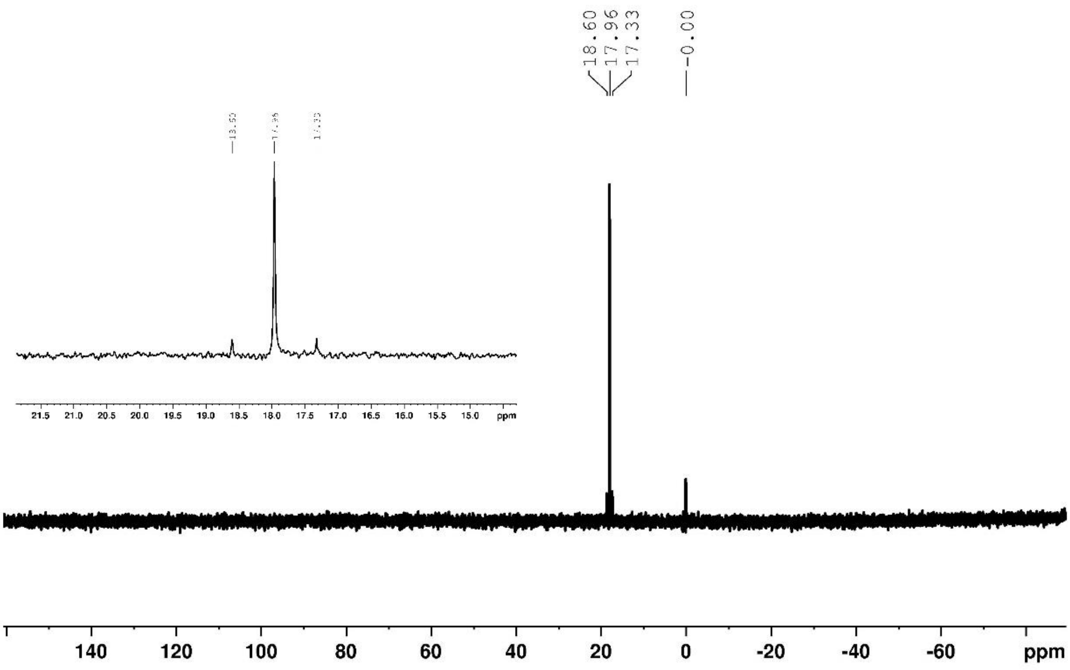

Figure S12 ^{29}Si-NMR (80 MHz, 25 °C, C_6D_6) spectra of **6**.

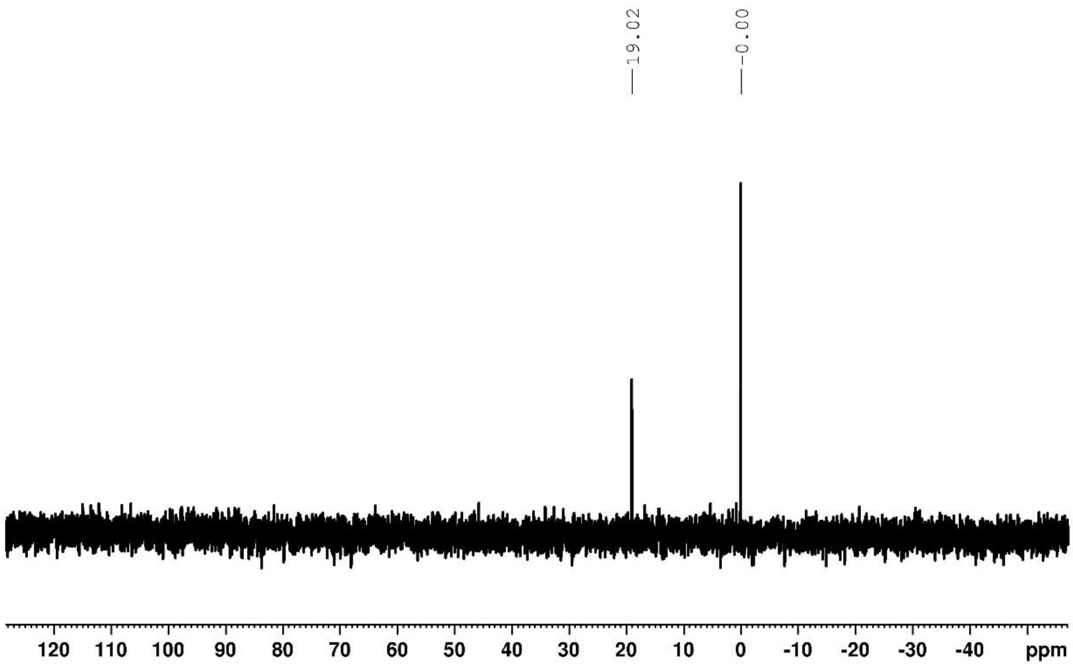

Figure S13 ^{29}Si-NMR (80 MHz, 25 °C, C_6D_6) spectra of **7**.

2.4. ^{77}Se NMR spectra

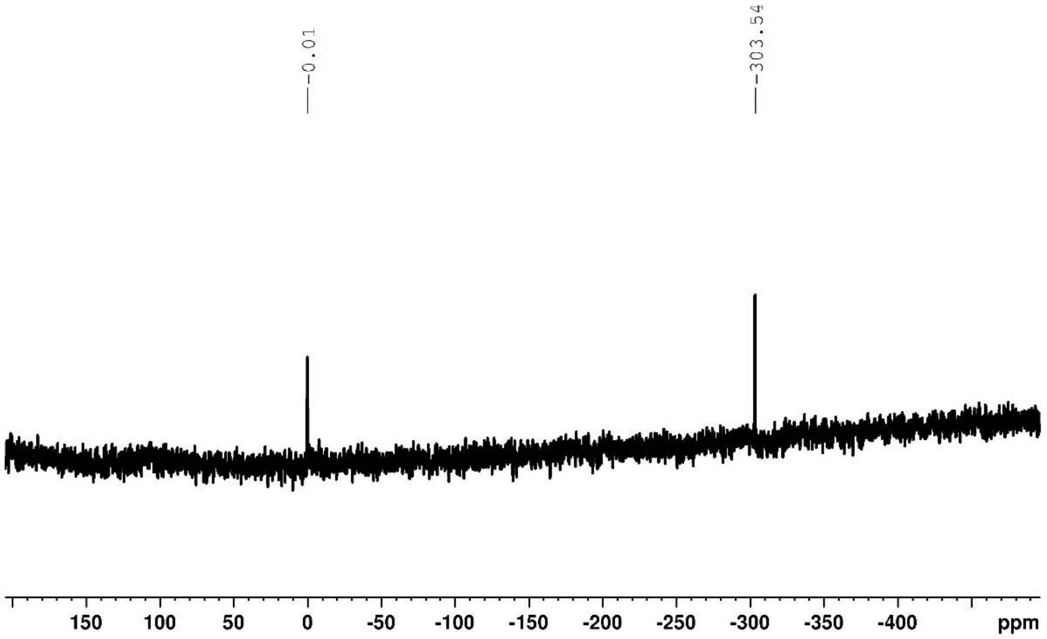

Figure S14 ^{77}Se-NMR (76 MHz, 25 °C, C_6D_6) spectra of **4**.

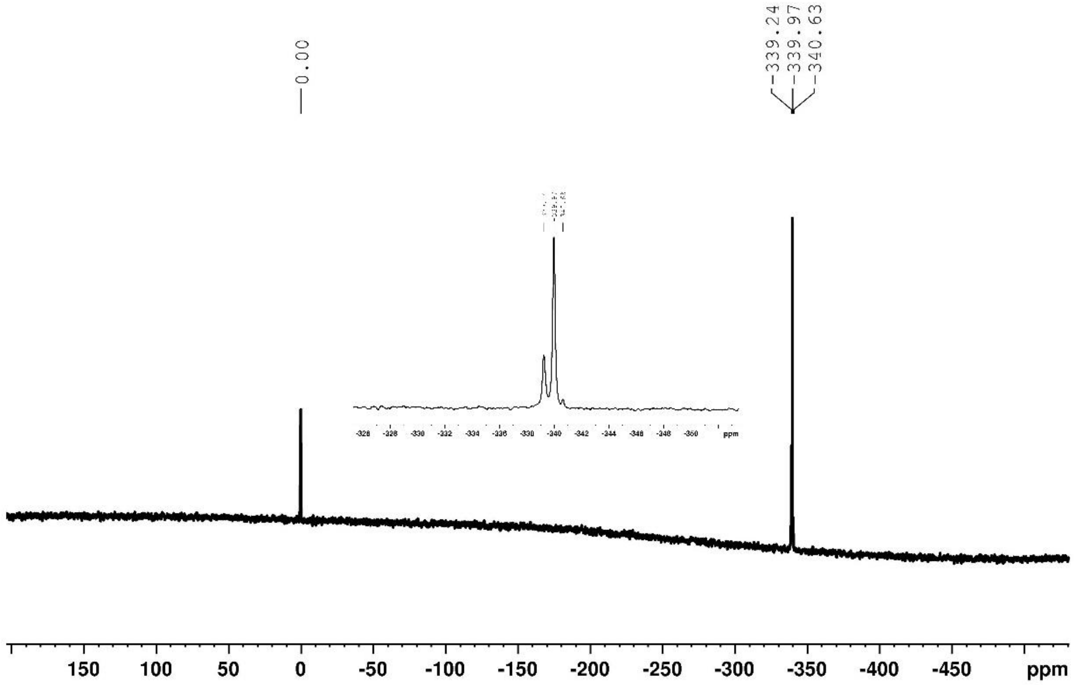

Figure S15 ^{77}Se-NMR (76 MHz, 25 °C, C_6D_6) spectra of **5** (mixture).

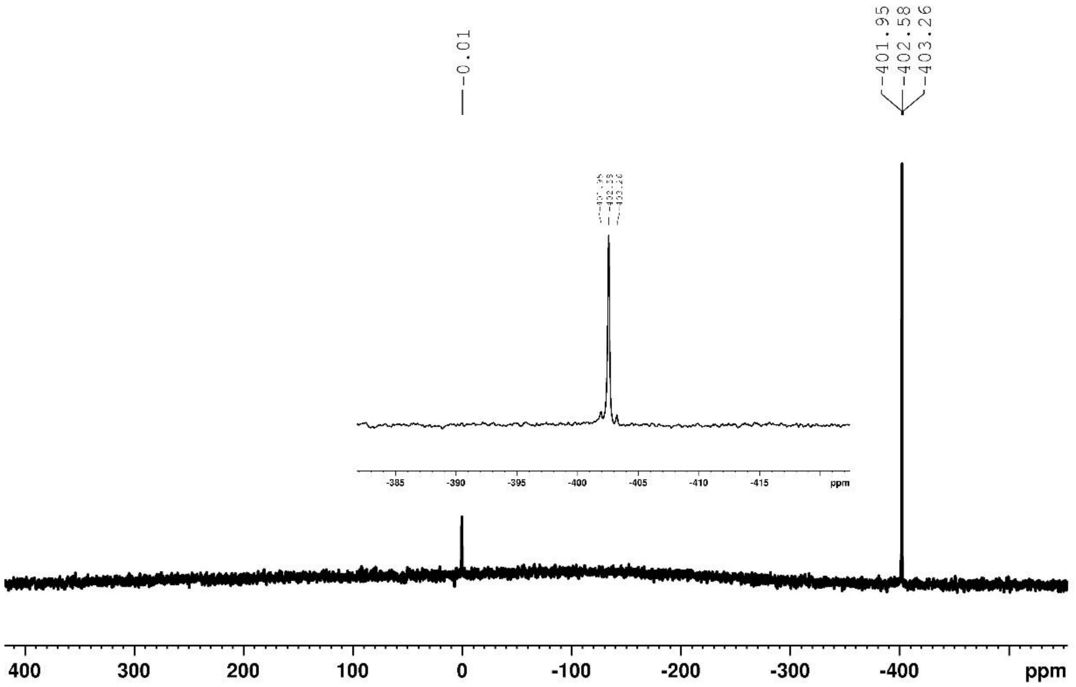

Figure S16 ^{77}Se-NMR (76 MHz, 25 °C, C_6D_6) spectra of **6**.

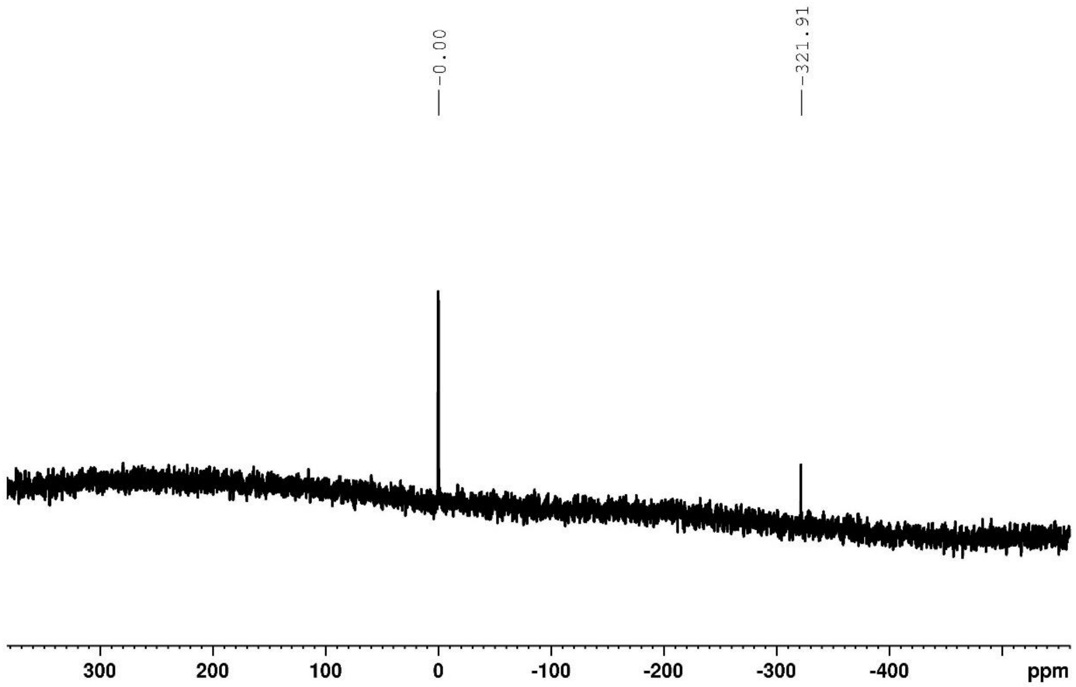

Figure S17 ^{77}Se-NMR (76 MHz, 25 °C, C_6D_6) spectra of **7**.

3. GC/MS records

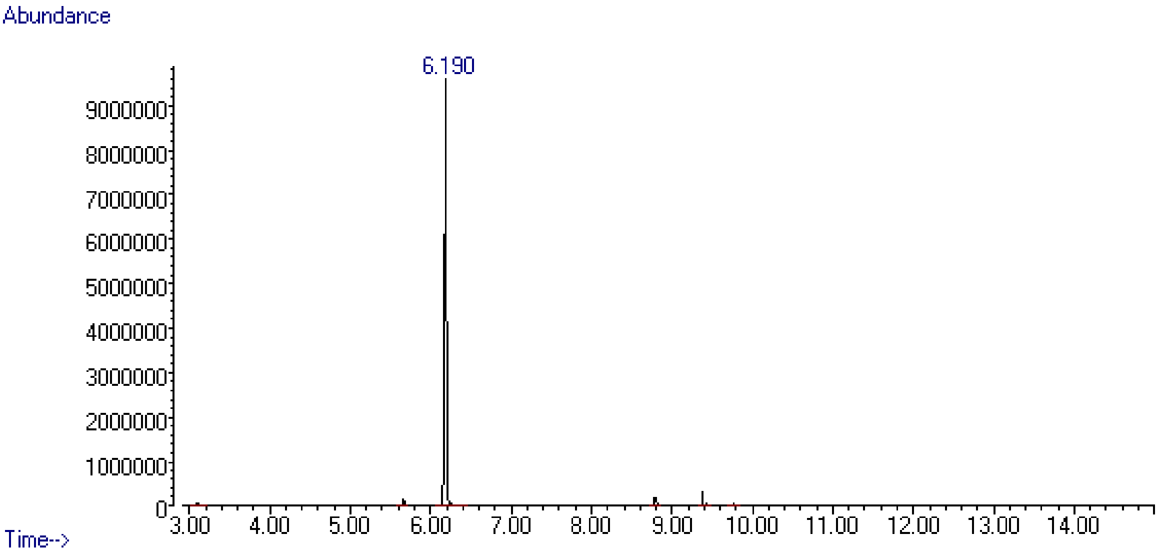

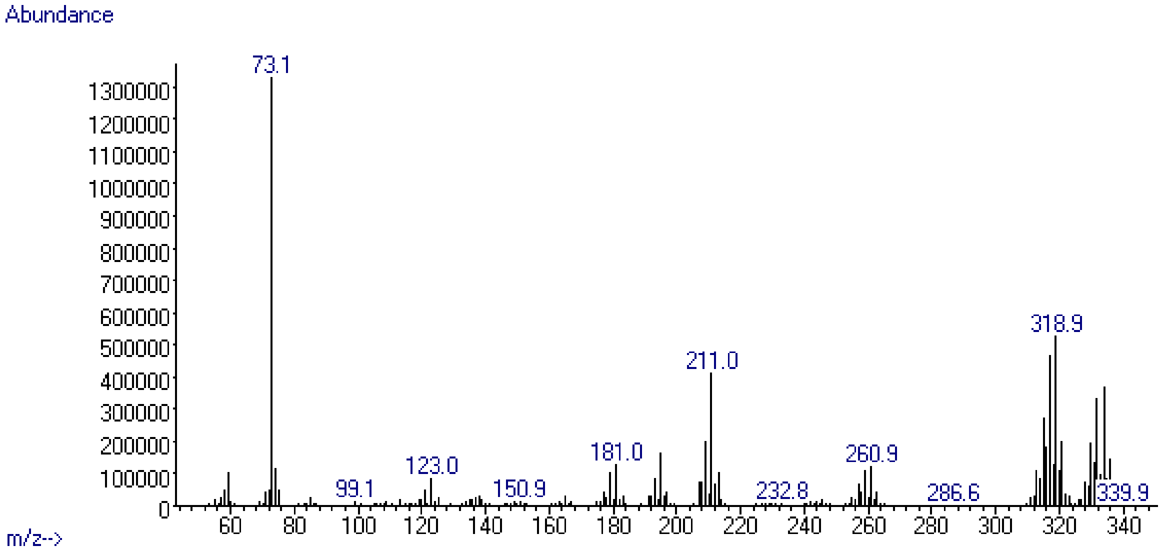

Figure S18 GC/MS record of **4**.

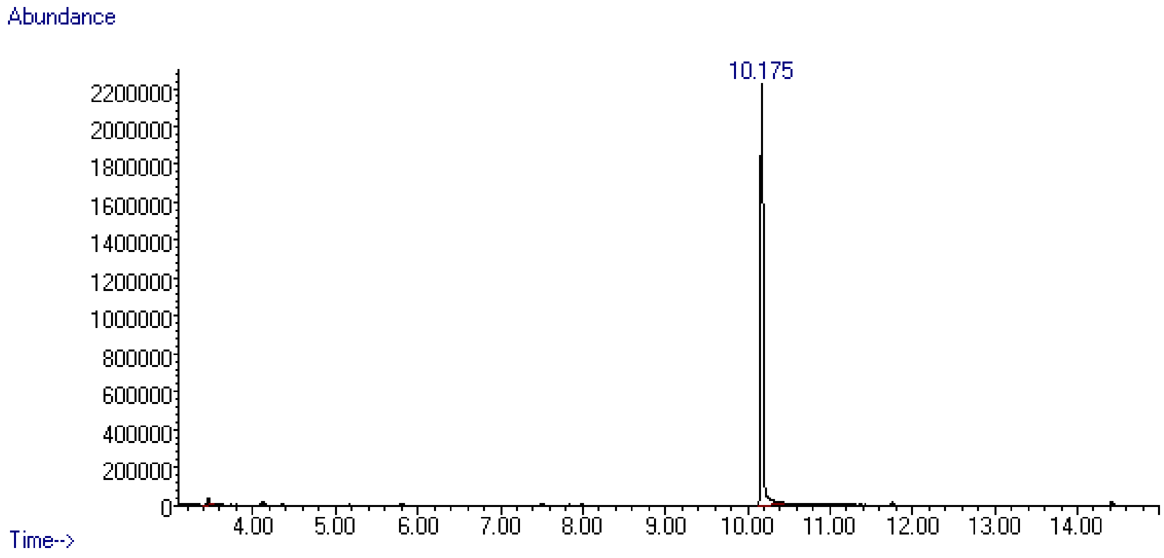

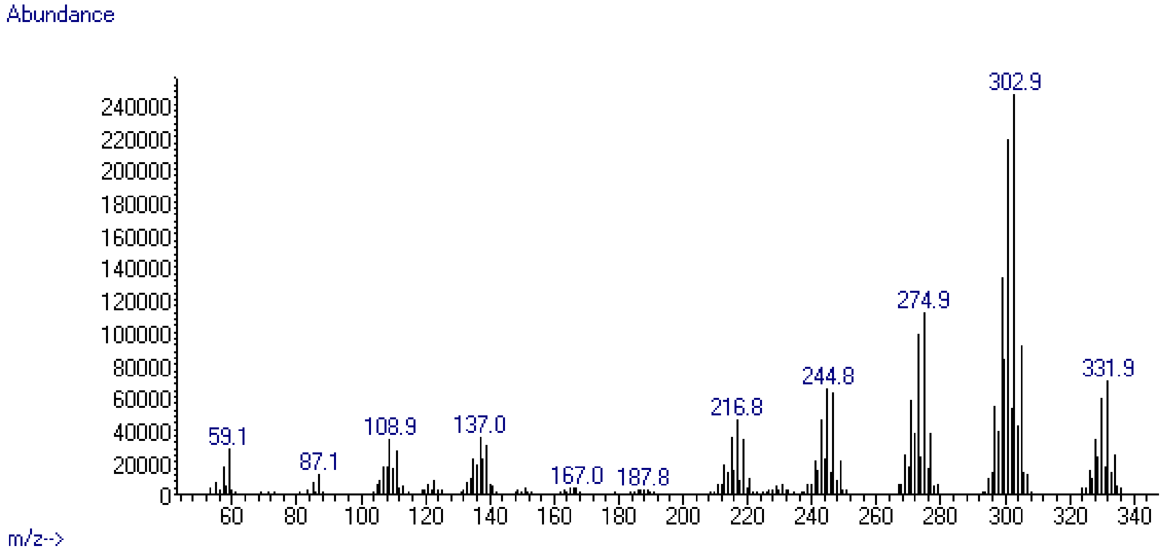

Figure S19 GC/MS record of **5** (mixture).

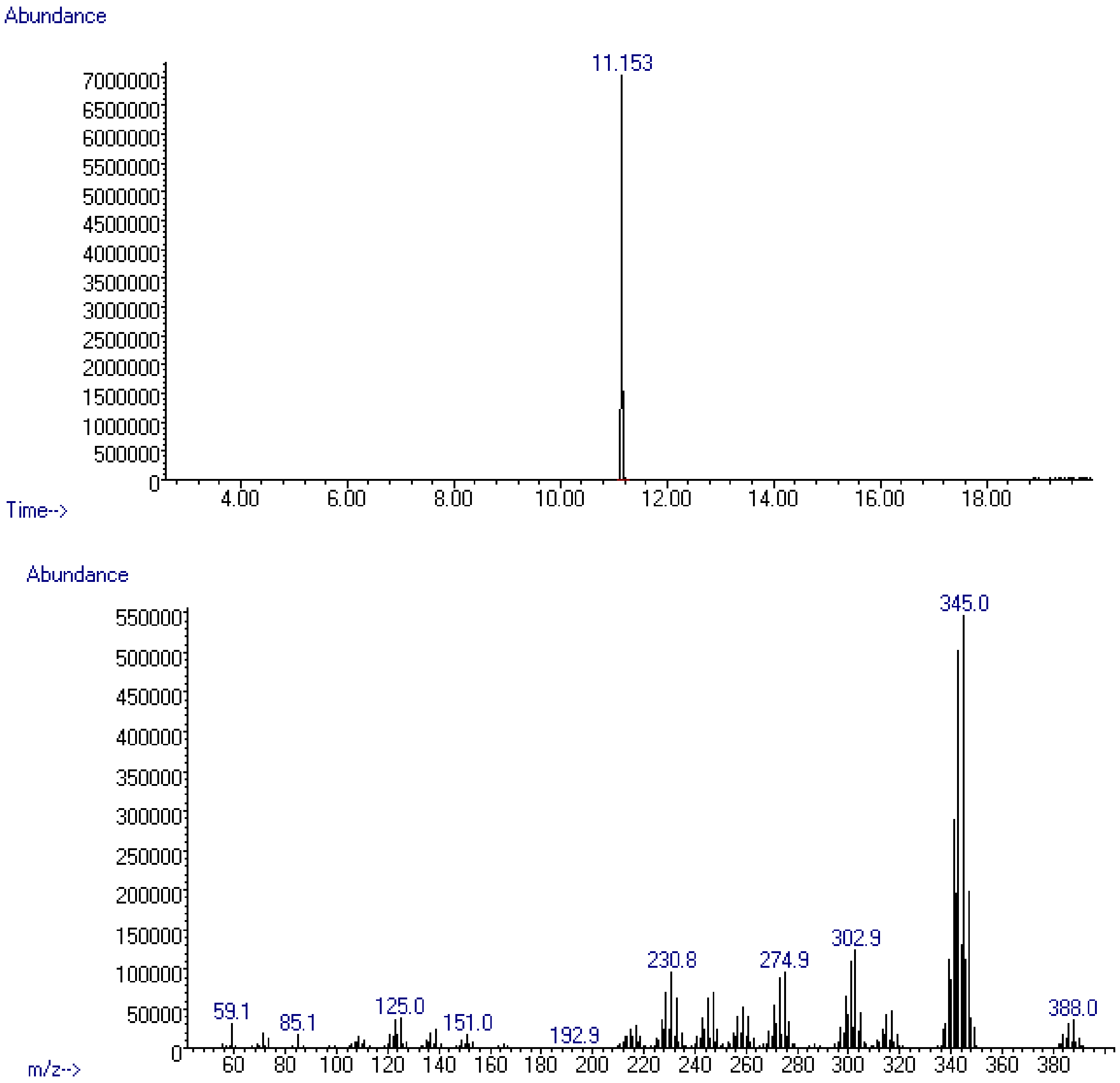

Figure S20 GC/MS record of **6**.

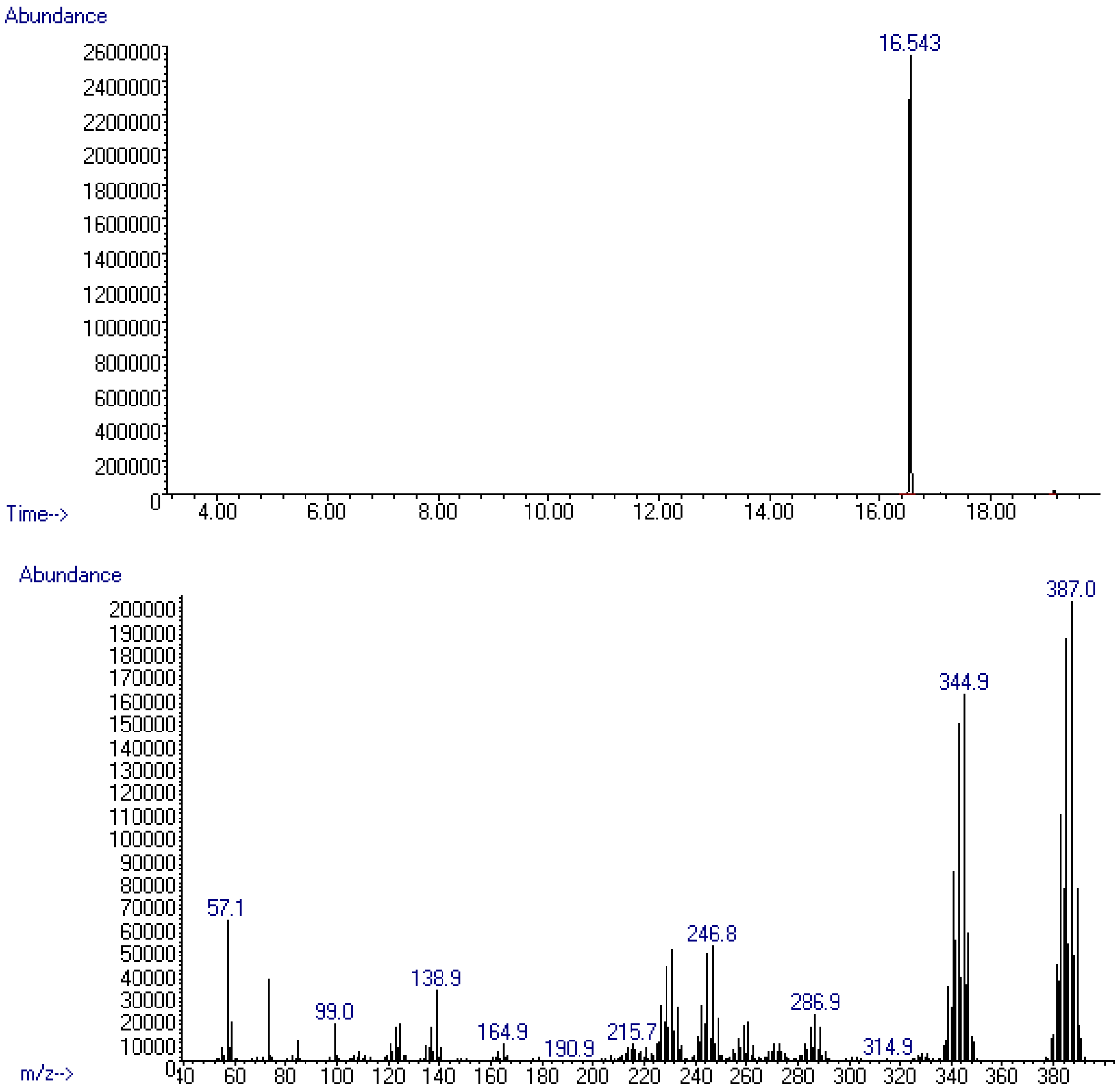

Figure S21 GC/MS record of **7**.

4. DSC thermograms

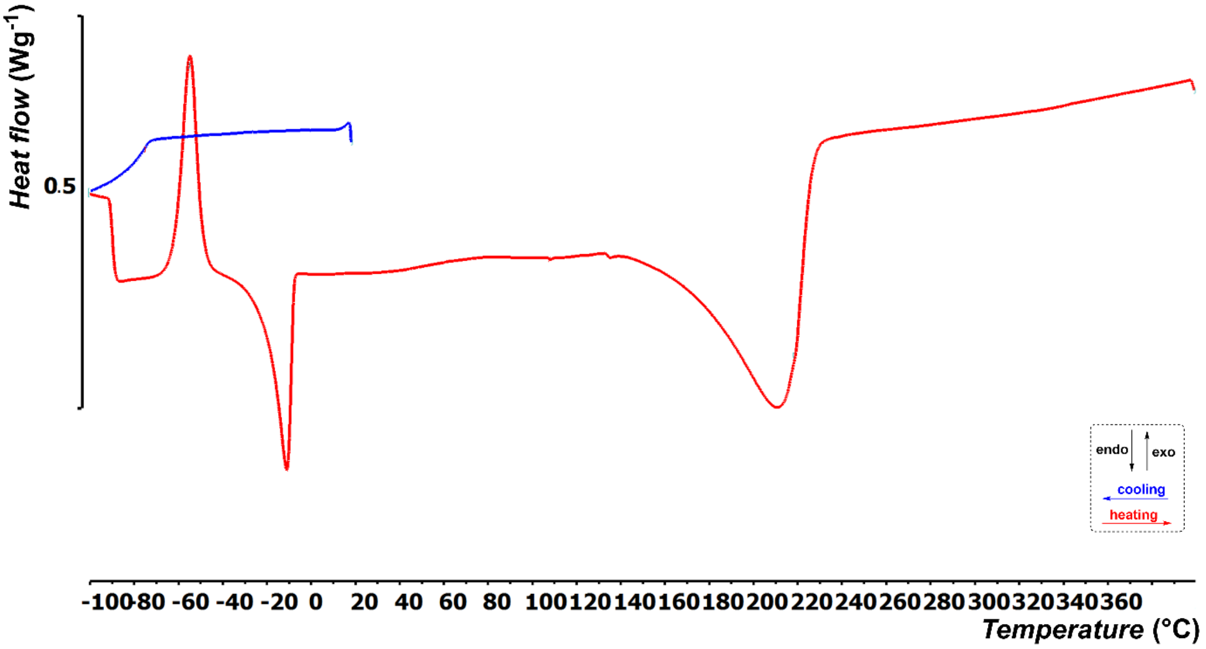

Figure S22 DSC curve of **4**.

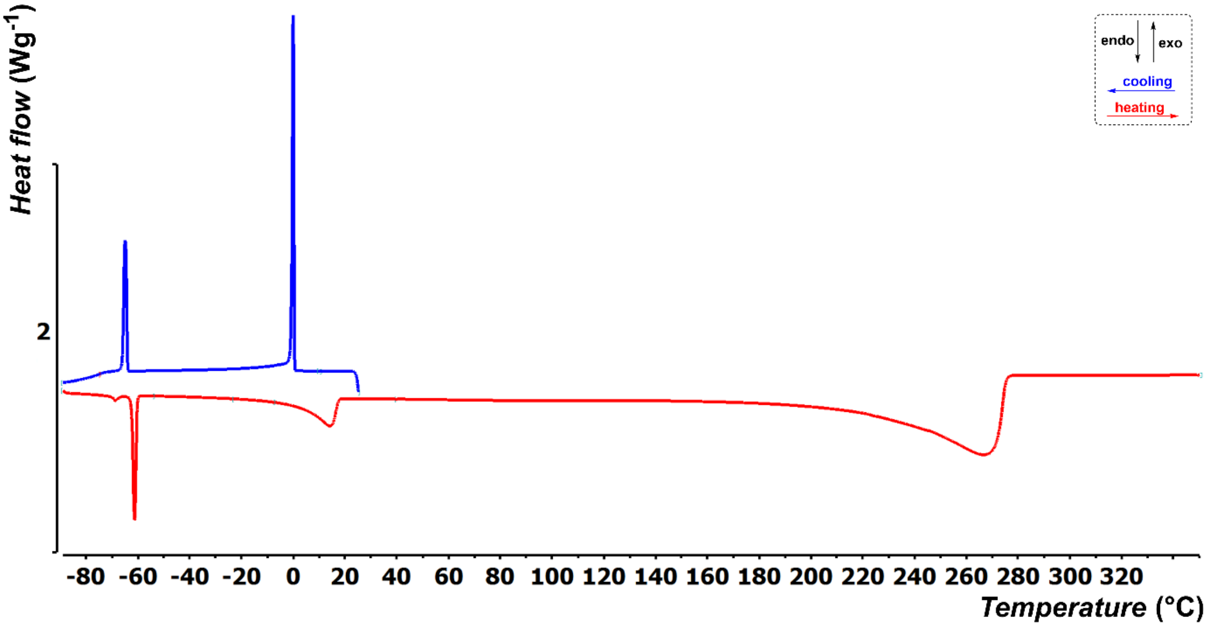

Figure S23 DSC curve of **5** (mixture)

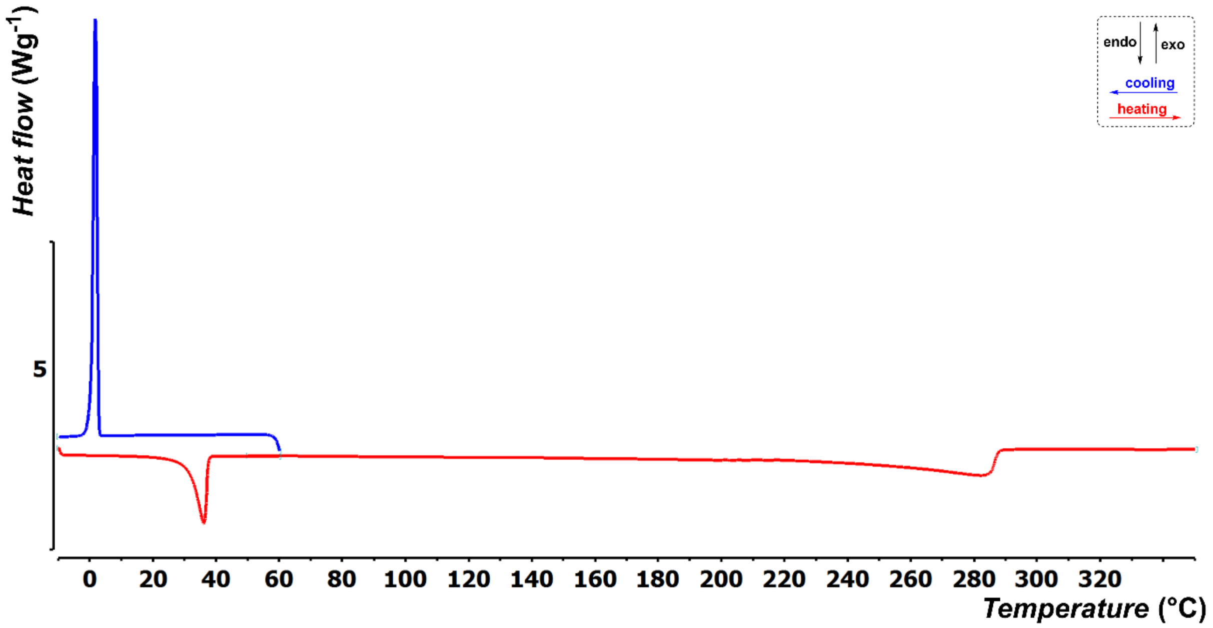

Figure S24 DSC curve of **6**.

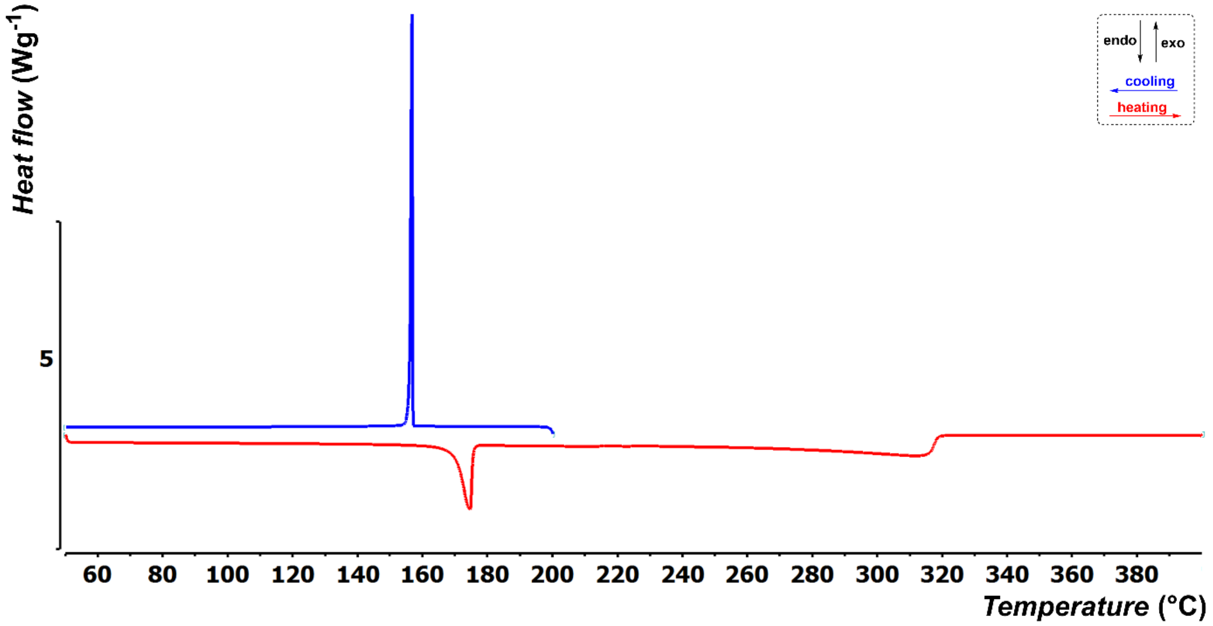

Figure S25 DSC curve of **7**.

5. TGA

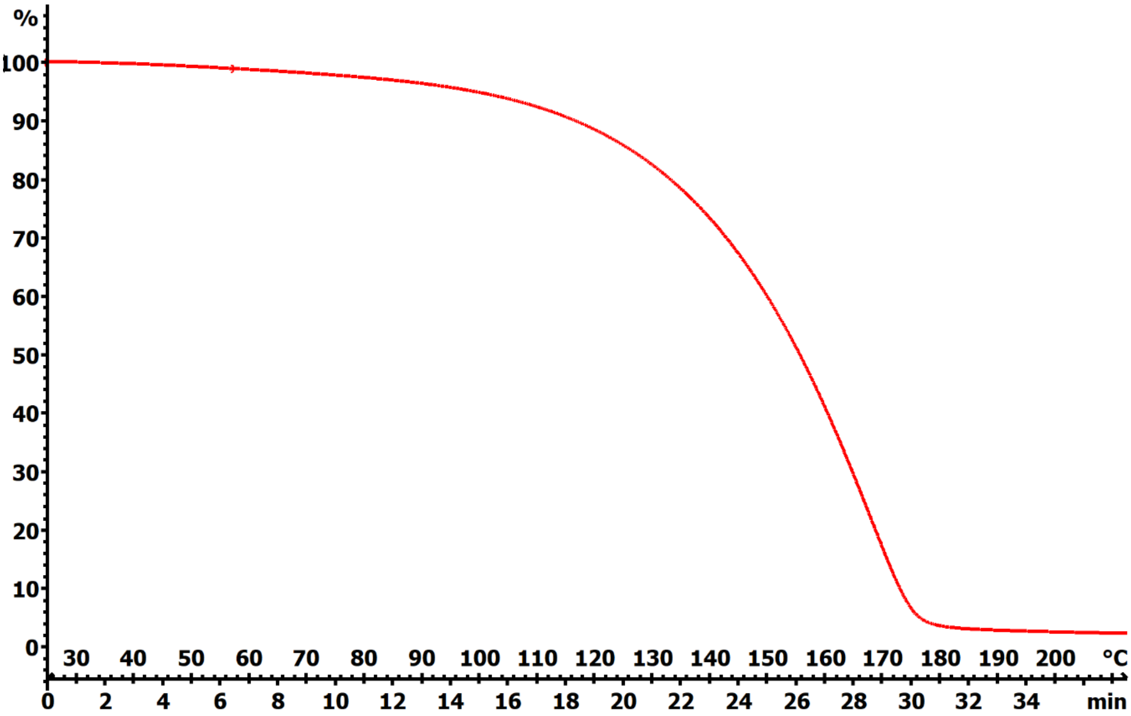

Figure S26 TGA curve of **4**.

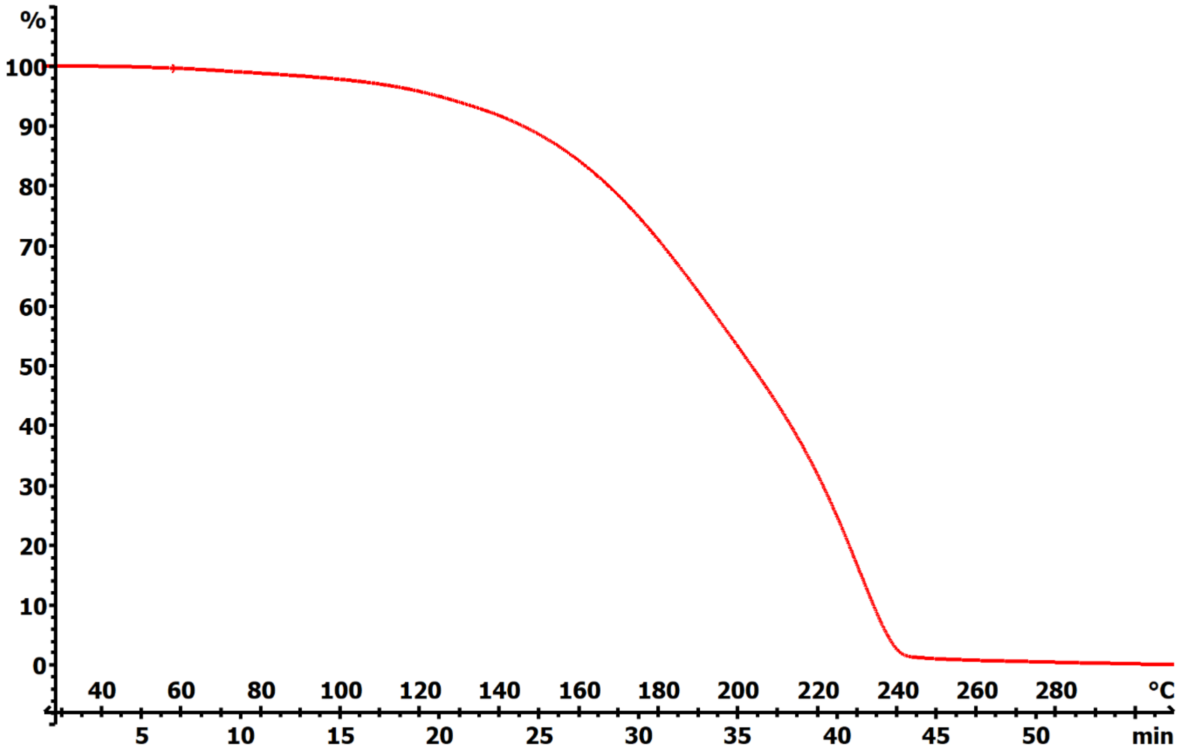

Figure S27 TGA curve of **5** (mixture).

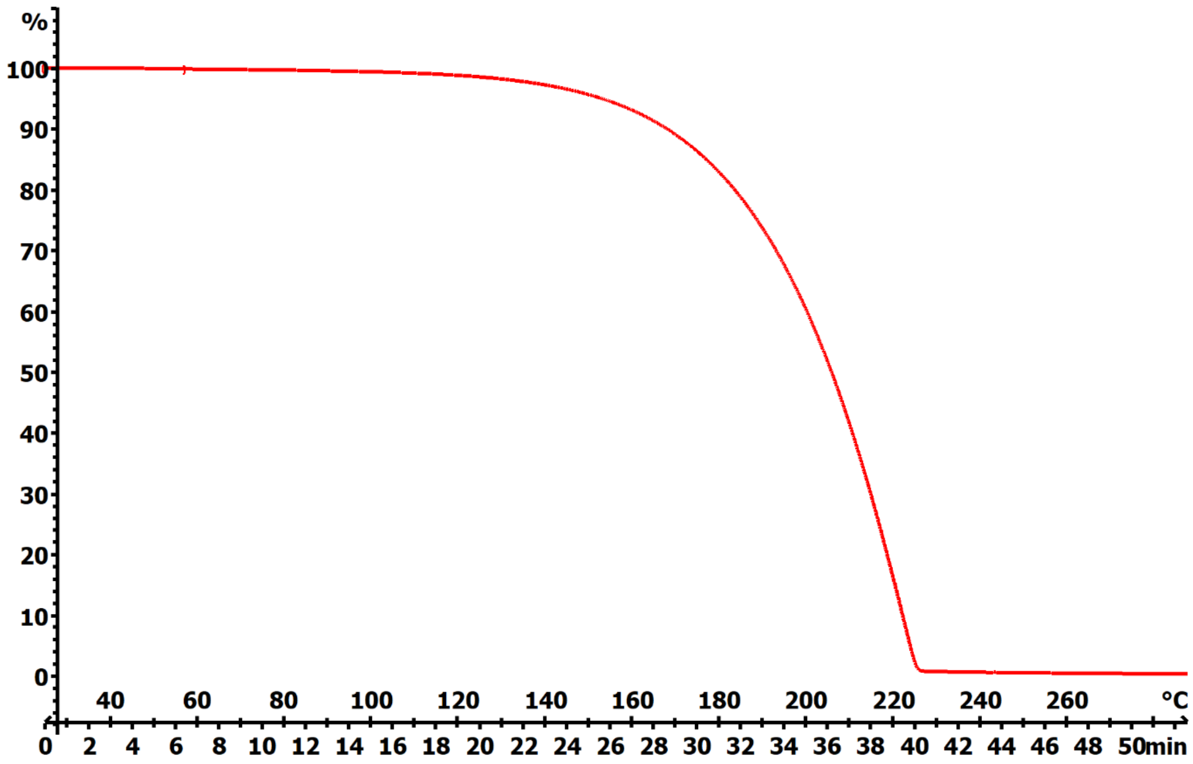

Figure S28 TGA curve of **6**.

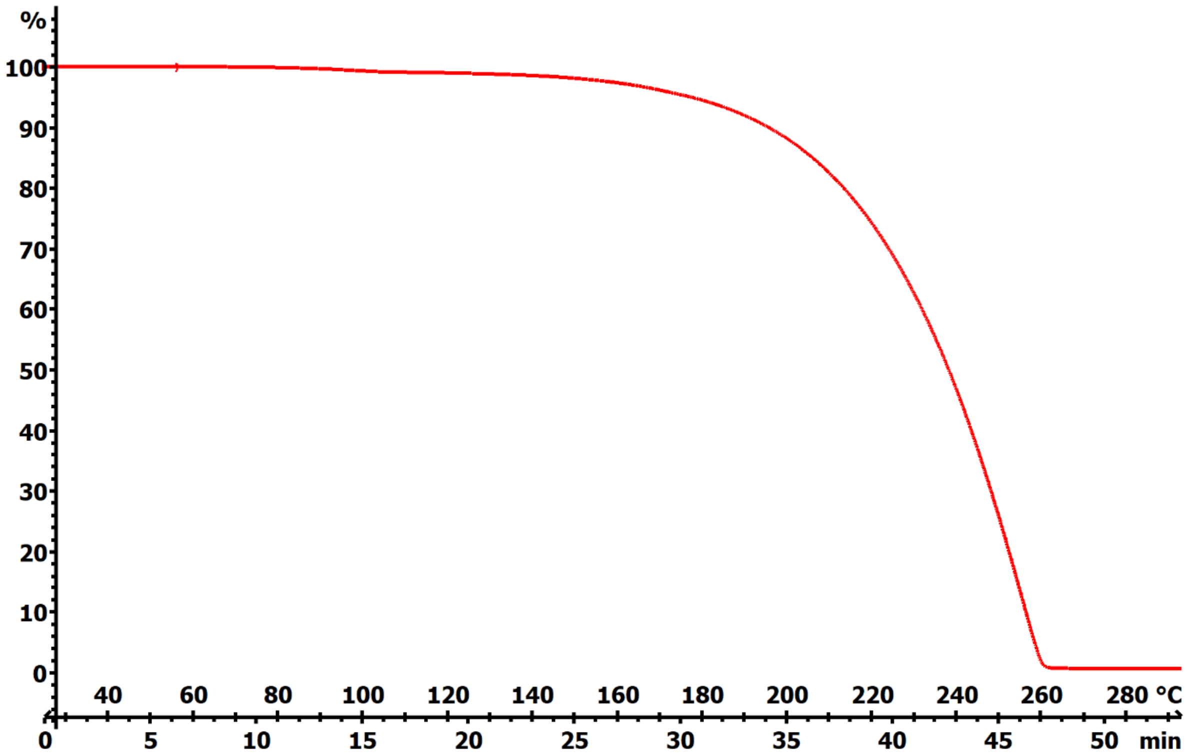

Figure S29 TGA curve of **7**.

6. SEM

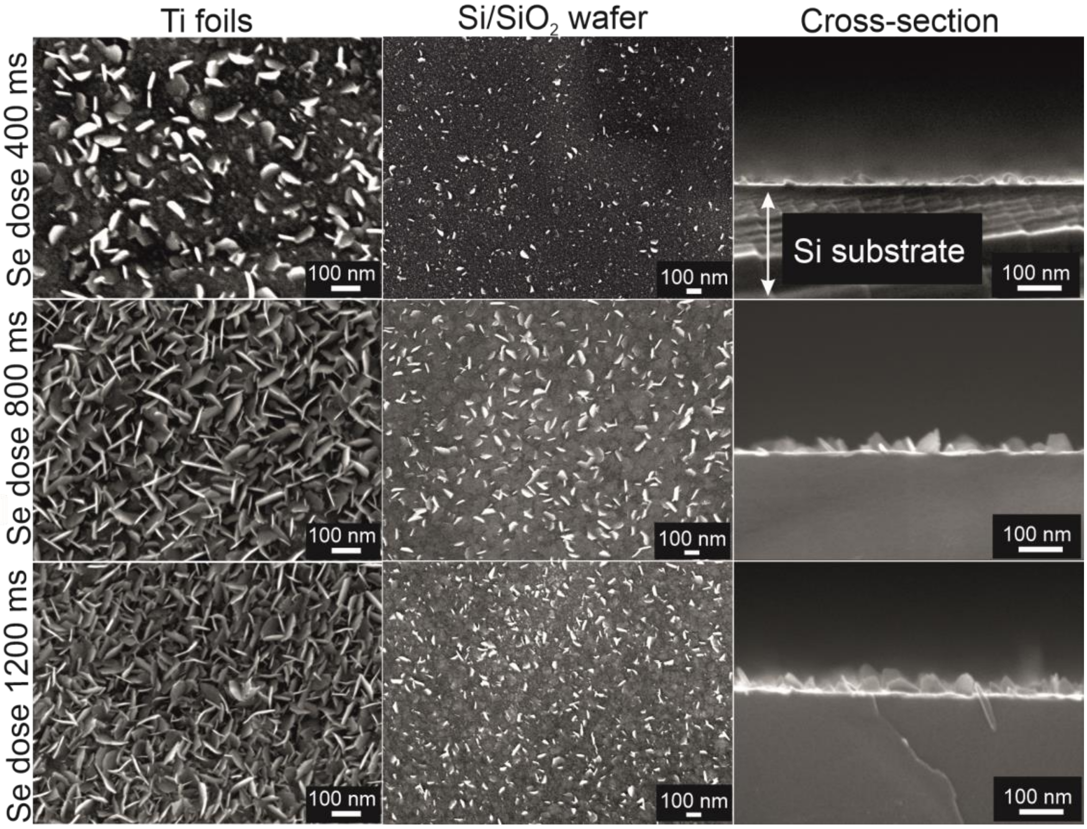

Figure S30 Left and central column: SEM top view images of as deposited $MoSe_2$ at 300°C using Se precursor **6** upon 800 ALD cycles on annealed titanium foil and silicon wafer, respectively, applying constant Mo dose (800 ms) and different Se dosing, namely, 400, 800 and 1200 ms; Right column: the cross-sectional SEM images of the Si wafers corresponding to samples from the central column.

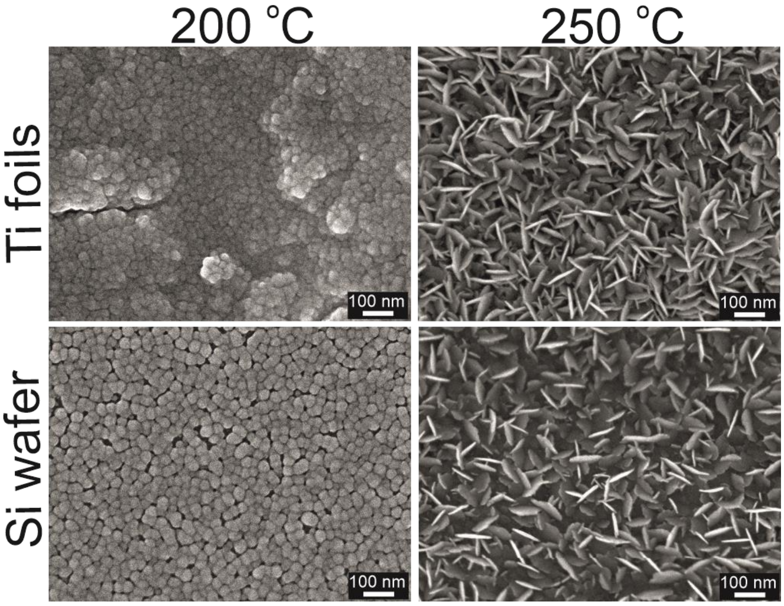

Figure S31 SEM top view images of $MoSe_2$ deposited at 200 and 250°C using Se precursor **6** on annealed titanium foil and silicon wafer (800 ms Se dose) upon 800 ALD cycles.

7. Raman spectroscopy

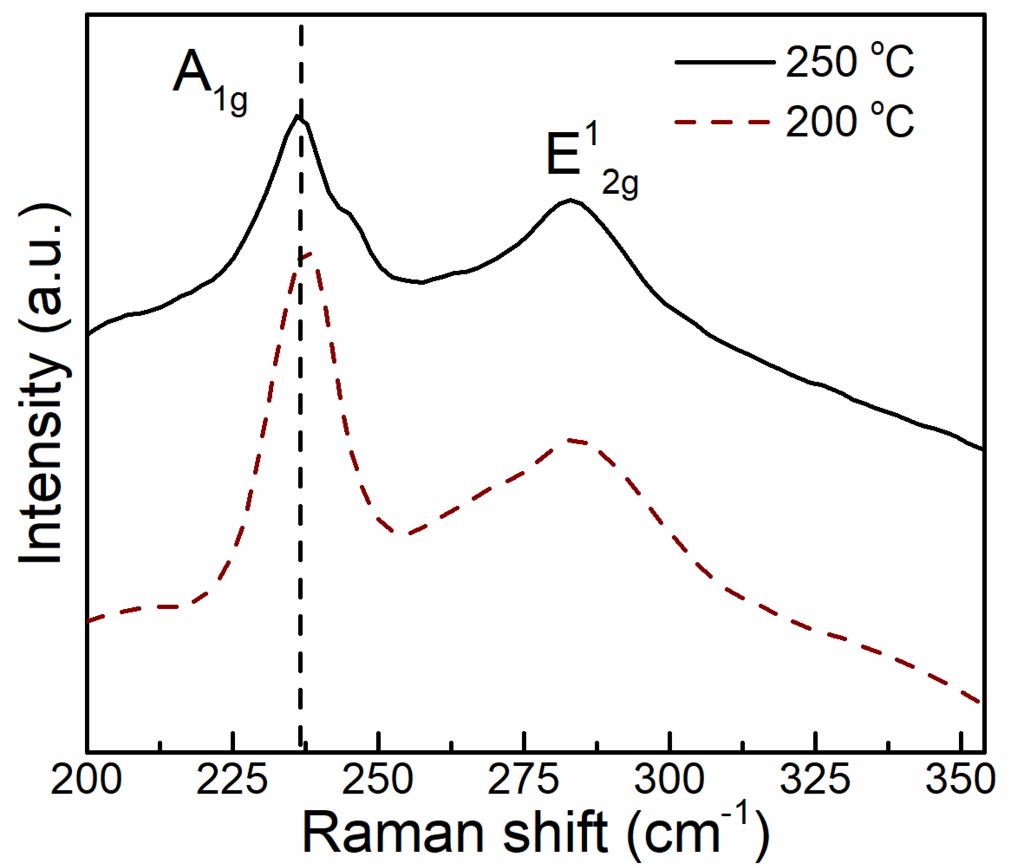

Figure S32 Raman spectra obtained from $MoSe_2$ deposited at 200 and 250°C using Se precursor **6** upon 800 ALD cycles on annealed titanium foil (800 ms Se dose).

8. XPS

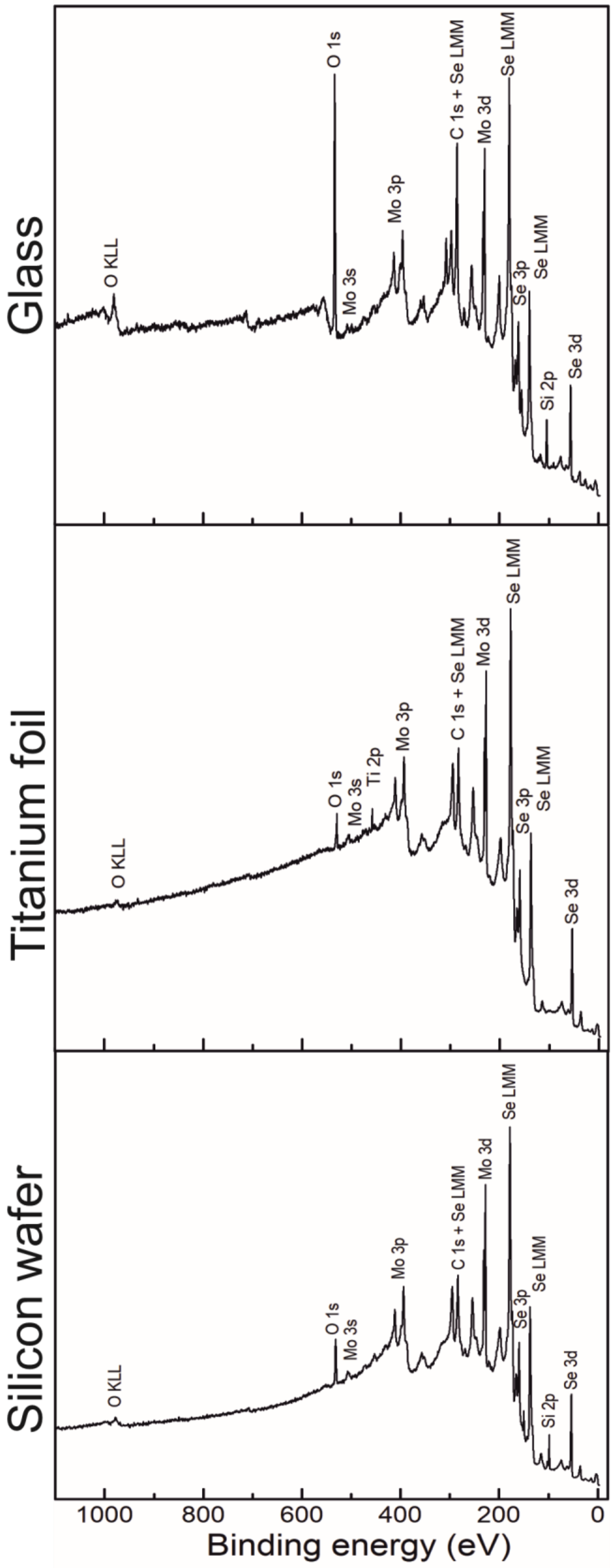

Figure S33 XPS survey spectra of ALD $MoSe_2$ deposited at 300 °C using Se precursor **6** upon 800 ALD cycles (800 ms Se dose) on different substrates: glass, titanium foil and silicon wafer.

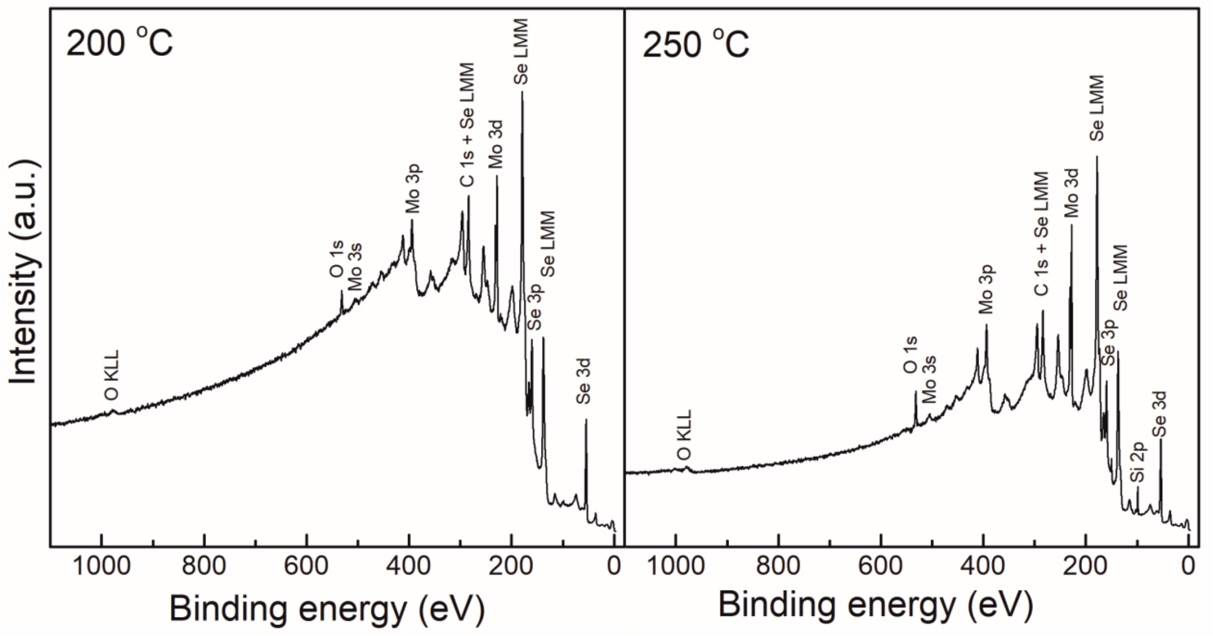

Figure S34 XPS survey spectra of ALD $MoSe_2$ deposited at 200 and 250 °C using Se precursor **6** upon 800 ALD cycles (800 ms Se dose) on silicon wafer.

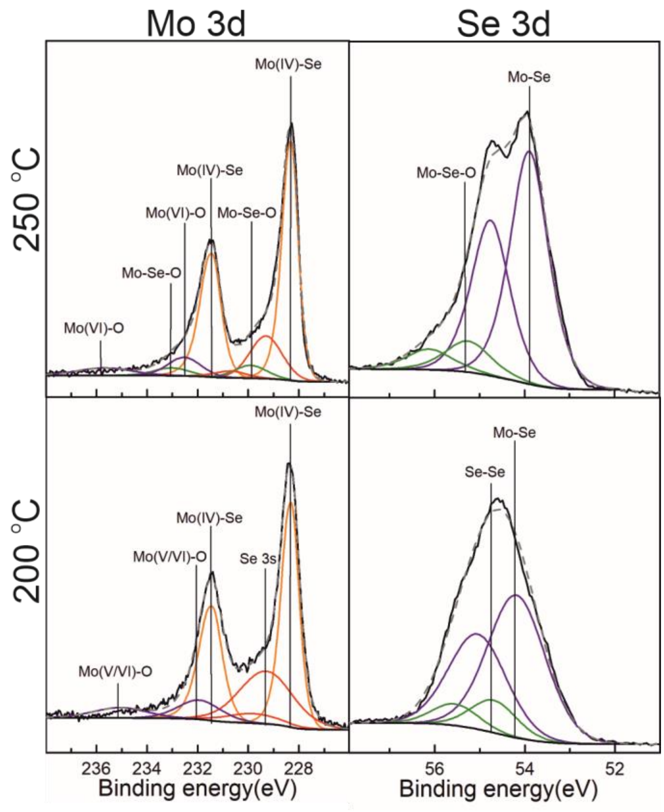

Figure 35 XPS high-resolution spectra of Mo 3d (left) and Se 3d (right) corresponding to ALD $MoSe_2$ (800 ms Se dose) deposited on silicon wafer at 200 and 250 °C upon 800 ALD cycles using Se precursor **6**.
